# Identification and validation of anti-protein arginine methyltransferase 5 (PRMT5) antibody as a novel biomarker for systemic sclerosis (SSc)

**DOI:** 10.1136/ard-2024-225596

**Published:** 2024-04-29

**Authors:** Minrui Liang, Lingbiao Wang, Xiaolong Tian, Kun Wang, Xiaoyi Zhu, Linlin Huang, Qing Li, Wenjing Ye, Chen Chen, Haihua Yang, Wanqing Wu, Xiangjun Chen, Xiaoxia Zhu, Yu Xue, Weiguo Wan, Yanling Wu, Liwei Lu, Jiucun Wang, Hejian Zou, Tianlei Ying, Feng Zhou

**Affiliations:** 1 Department of Rheumatology, Huashan Hospital, Fudan University, Shanghai, China; 2 Institute of Rheumatology, Immunology and Allergy, Fudan University, Shanghai, China; 3 Huashan Rare Disease Center, Huashan Hospital, Fudan University, Shanghai, China; 4 Key Laboratory of Medical Molecular Virology (MOE/NHC/CAMS) and Shanghai Institute of Infectious Disease and Biosecurity, Shanghai Frontiers Science Center of Pathogenic Microorganisms and Infection, Shanghai Engineering Research Center for Synthetic Immunology, School of Basic Medical Sciences, Fudan University, Shanghai, China; 5 Shanghai Key Laboratory of Medical Epigenetics, International Co-laboratory of Medical Epigenetics and Metabolism, Ministry of Science and Technology, Institutes of Biomedical Sciences, Fudan University, Shanghai, China; 6 Key Laboratory of Carcinogenesis and Cancer Invasion, Ministry of Education, Liver Cancer Institute, Zhongshan Hospital, Fudan University, Shanghai, China; 7 Department of Emergency Medicine, Zhongshan Hospital, Fudan University, Shanghai, China; 8 Department of Respiratory and Critical Care Medicine, Huashan Hospital, Fudan University, Shanghai, China; 9 Department of Neurology, Huashan Hospital, Fudan University, Shanghai, China; 10 Department of Pathology, The University of Hong Kong, Hong Kong, China; 11 State Key Laboratory of Genetic Engineering, Collaborative Innovation Center for Genetics and Development, School of Life Sciences, and Human Phenome Institute, Fudan University, Shanghai, China

**Keywords:** Autoantibodies, Autoimmune Diseases, Scleroderma, Systemic

## Abstract

**Objectives:**

In the complex panorama of autoimmune diseases, the characterisation of pivotal contributing autoantibodies that are involved in disease progression remains challenging. This study aimed to employ a global antibody profiling strategy to identify novel antibodies and investigate their association with systemic sclerosis (SSc).

**Methods:**

We implemented this strategy by conducting immunoprecipitation (IP) following on-bead digestion with the sera of patients with SSc or healthy donors, using antigen pools derived from cell lysates. The enriched antigen-antibody complex was proceeded with mass spectrometry (MS)-based quantitative proteomics and over-represented by bioinformatics analysis. The candidate antibodies were then orthogonally validated in two independent groups of patients with SSc. Mice were immunised with the target antigen, which was subsequently evaluated by histological examination and RNA sequencing.

**Results:**

The IP-MS analysis, followed by validation in patients with SSc, revealed a significant elevation in anti-PRMT5 antibodies among patients with SSc. These antibodies exhibited robust diagnostic accuracy in distinguishing SSc from healthy controls and other autoimmune conditions, including systemic lupus erythematosus and Sjögren’s syndrome, with an area under the curve ranging from 0.900 to 0.988. The elevation of anti-PRMT5 antibodies was verified in a subsequent independent group with SSc using an additional method, microarray. Notably, 31.11% of patients with SSc exhibited seropositivity for anti-PRMT5 antibodies. Furthermore, the titres of anti-PRMT5 antibodies demonstrated a correlation with the progression or regression trajectory in SSc. PRMT5 immunisation displayed significant inflammation and fibrosis in both the skin and lungs of mice. This was concomitant with the upregulation of multiple proinflammatory and profibrotic pathways, thereby underscoring a potentially pivotal role of anti-PRMT5 antibodies in SSc.

**Conclusions:**

This study has identified anti-PRMT5 antibodies as a novel biomarker for SSc.

**WHAT IS ALREADY KNOWN ON THIS TOPIC**

- The involvement of autoantibodies in the development of systemic sclerosis (SSc), particularly those not linked to well-defined autoantigens, remains largely unknown.
- SSc-specific autoantibodies play a critical role in the diagnosis, differentiation and stratification of the disease.
- Ongoing efforts are underway to discover novel autoantibodies using advanced techniques.

**WHAT THIS STUDY ADDS**

- This study represents the initial identification and validation of anti-PRMT5 antibodies in two independent groups of patients with SSc.
- Levels of anti-PRMT5 antibodies exhibited a correlation with the disease trajectory in SSc, serving as a predictive indicator for regression or progression in both skin and lung involvement.
- Immunisation with recombinant protein PRMT5 induced SSc-like manifestations in mice, indicating a potentially pivotal role in SSc.

**HOW THIS STUDY MIGHT AFFECT RESEARCH, PRACTICE OR POLICY**

- Anti-PRMT5 antibodies manifested as a diagnostic and predictive marker for SSc.
- The application of anti-PRMT5 antibodies may contribute to precisive disease monitoring and prognosis.

## Introduction

Systemic sclerosis (SSc) is an autoimmune rheumatic disease, characteristic of autoimmunity with increased inflammatory burden, vasculopathy with extensive endothelial dysfunction and tissue fibrosis with fibroblast activation.^1–4^ An abundance of autoantibodies detected in SSc and the close link with clinical outcomes indicate the potential involvement of autoantibodies and the breakdown of self-tolerance in the pathogenesis of SSc, thereby offering up important novel diagnostic and therapeutic opportunities of autoantibodies.^5 6^ Nevertheless, the well-defined SSc autoantigens are ubiquitously expressed and play an essential role in physiological processes. Consequently, the strong association of specific SSc autoantibodies with clinical phenotypes raises intriguing questions regarding their roles in SSc pathogenesis: are these autoantibodies the drivers or merely incidental bystanders?

Antitopoisomerase antibodies (ATAs), anticentromere antibodies (ACAs) and anti-RNA polymerase III antibodies (ARAs), three types of antinuclear antibodies (ANAs), have been reported as the most prevalent SSc-specific antibodies. However, up to 11% of patients with SSc are negative for ANA,^7^ and even as many as 17% of patients with SSc lack detectable levels of established SSc-specific antibodies.^8^ This highlights the necessity to explore novel autoantibodies that are specific to SSc. There is a growing body of studies focusing on the identification of novel autoantibodies and the definition of their clinical implications, such as antiplatelet-derived growth factor receptor (PDGFR), antiangiotensin receptor type 1, antigephyrin and antieukaryotic initiation factor 2B antibodies.^9–14^ Mechanistically, single-cell analysis reveals broad differences in cell cluster gene expression profiles, showing distinctions in clinical phenotypes and distinct skin score trajectories across autoantibody subgroups of diffuse cutaneous SSc (dcSSc).^15^ However, further investigation is required to elucidate the precise pathomechanism of autoantibodies in SSc.

Advanced high-profile techniques, such as solid surface arrays and display technologies,^16^ have been boosting the biomarker discovery. Despite considerable progress and widespread application in autoantibody discovery,^17–19^ the utility of high-throughput assays, particularly protein microarrays, remains restricted by the limited amount of miniaturised test sites. This constrains the coverage of the whole proteome and the detection sensitivity and specificity for individual protein. In addition, patient heterogeneity and varied abundance of targets can limit the progress in finding specific biomarkers in serum. Therefore, a comprehensive strategy with pre-enrichment of protein targets followed by subsequent whole-proteome screening for functional properties will facilitate the investigation of serum alterations in patients.^20 21^ However, this approach necessitates extensive proof-of-concept studies and wide-spectrum cohort validation.

Here, we first identified PRMT5 as a novel autoantibody target in SSc based on the automated deep efficient peptide sequencing and quantification (DEEP SEQ) mass spectrometry (MS) platform^22^ for the enriched antigen-antibody complex. We then validated the prevalence of antibodies against PRMT5 in the sera of patients with SSc and demonstrated the close correlations of anti-PRMT5 antibodies with the progression or regression trajectories of skin and lung disease in SSc. Induction of SSc-like skin and lung changes in mice via immunisation with recombinant PRMT5 protein indicates anti-PRMT5 as a potential contributing antibody in the pathogenesis of SSc. Anti-PRMT5 antibodies manifested as diagnostic and predictive marker for SSc. The utilisation of anti-PRMT5 antibodies has the potential to enhance precise disease monitoring and prognosis assessment in SSc.

## Result

### Global autoantibody profiling in SSc via DEEP SEQ proteomics

**Figure 1:**
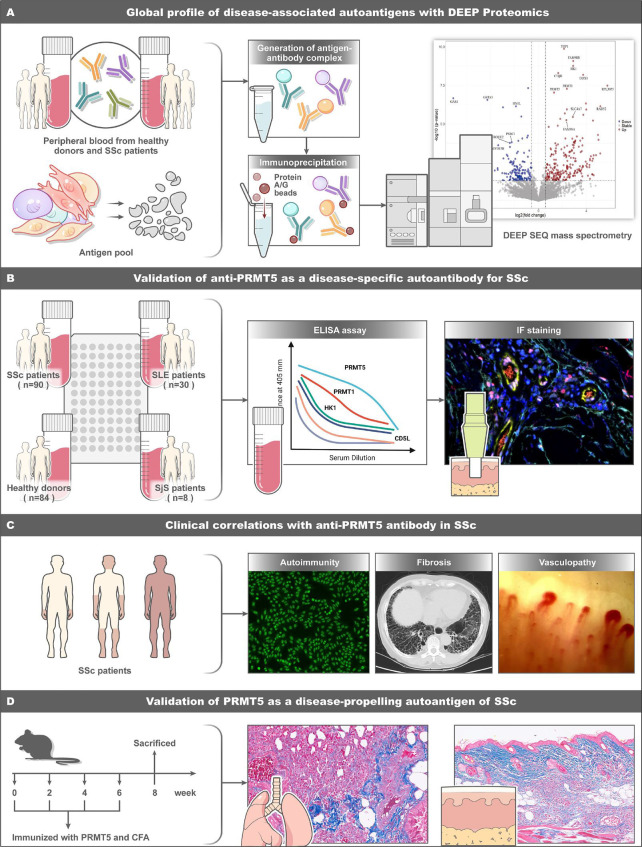
Overview of experimental workflow and key discoveries. (A) Peripheral blood was obtained from the patients with SSc and healthy subjects (n=3/group), and then coincubated with an antigen pool, which was prepared from a diverse array of cell lysate of endothelial cells, fibroblasts, T cells and monocytes. Then the antigen-antibody complex was enriched with protein A/G and proceeded with on-bead digestion and labelling by means of isobaric tags for relative and absolute quantitation (iTRAQ). The quantitative proteomics of these pulldowns enabled us to illuminate the presence of over-represented putative antibody targets in patients. Applying the automated deep efficient peptide sequencing and quantification (DEEP SEQ) mass spectrometry (MS) platform,^37^ our immunoprecipitation (IP)-MS analysis revealed putative autoantibodies against antigen pools, which require further verification to exclude non-specific binding. (B) Peripheral blood was obtained from 90 patients with systemic sclerosis (SSc) patients, 30 patients with systemic lupus erythematosus (SLE), 8 patients with Sjögren’s syndrome (SjS) and 84 healthy donors for antibody validation. Antibodies against PRMT1, PRMT5, HK1 and CD5L in the serum were determined by ELISA. Anti-PRMT5 was identified as a specific autoantibody for SSc. The expression of PRMT5 in SSc skin was evaluated by immunofluorescence (IF) staining. (C) The levels of anti-PRMT5 antibodies were compared between healthy subjects and patients with SSc and correlated with clinical phenotypes of patients with SSc. (D) Immunisation of mice with recombinant protein PRMT5 subcutaneously at an interval of 2 weeks for a total of four times. Skin and lung tissues were collected for pathological examination and bulk RNA sequencing (RNA-seq). Skin and lung fibrosis were found in PRMT5-immunised mice, which mimicked human SSc-like changes; thus, anti-PRMT5 antibody was identified as a potential contributing antibody for SSc.

**Figure 2:**
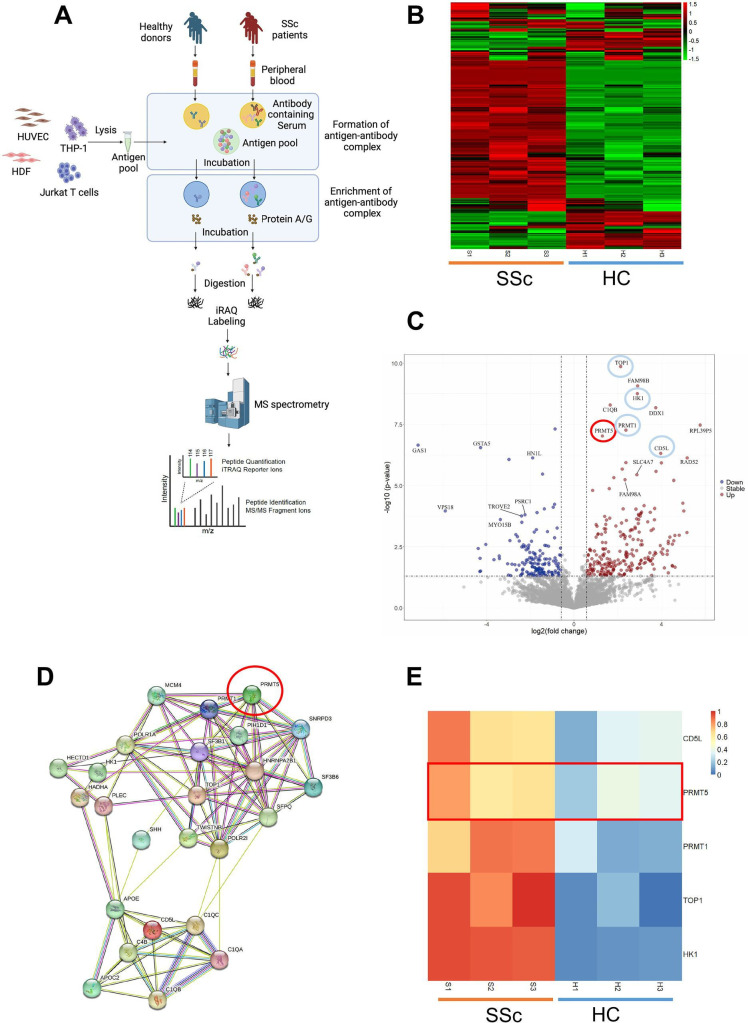
Proteomics-based discovery of autoantibodies in SSc. (A) Schematic illustration of the proteomics-based approach employed to identify autoantibodies associated with SSc. The graphic was generated using BioRender.com and complies with BioRender’s Academic License Terms. (B) Heatmap diagram displaying differentially expressed antibodies in the sera of patients with SSc compared with healthy controls. Red values indicate upregulation, while green values indicate downregulation. (C) Identification of autoantibodies by proteomics in SSc. Volcano plots illustrate iTRAQ proteomics results for enriched and purified proteins bound to antibodies in serum from patients with SSc versus healthy donors. The x-axis represents relative protein levels (mean log2 iTRAQ ratios across three replicate experiments) in patients with SSc compared with healthy donors, while the y-axis displays log10 (p values). Significantly enriched and upregulated proteins (p≤0.05; iTRAQ ratio ≥1.5) are denoted by red dots, significantly enriched and downregulated proteins (p≤0.05; iTRAQ ratio ≤−1.5) by blue dots, and all others by grey dots. The dotted lines indicate a 1.5-fold ratio (x-axis) and a p-value of 0.05 (y-axis). (D) Protein-protein interaction networks of a subset of significantly enriched and upregulated antibodies in SSc, highlighting PRMT5 as the antibody target. (E) Heatmap diagram depicting representative differentially expressed antibodies in SSc. Red values indicate upregulated proteins, while blue values indicate downregulated proteins. PRMT5 as the target is highlighted. iTRAQ, isobaric tags for relative and absolute quantitation; HC, healthy control; MS, mass spectrometry; SSc, systemic sclerosis.

To uncover elusive autoantibody surrogates for SSc, we developed a global antibody profiling strategy based on the automated DEEP SEQ MS platform^22^ (figures 1A and 2A). In brief, we constructed an antigen pool using lysates from a variety of cell lines, including human umbilical vein endothelial cells, human dermal fibroblasts, Jurkat T cells and THP-1 monocytes, as sources of antigens for subsequent proteomics profiling. The corresponding cell types have been linked to the pathogenesis of SSc.^4 13 14^ Antigen-antibody complexes were then enriched by coincubating global antibodies with the antigen pool, in conjunction with immunoprecipitation (IP) using protein A/G beads. Finally, we performed in-solution on-bead trypsin digest of the complexes and labelled peptide fragments, following subsequent quantitative proteomics analysis (figure 2A).

In this study, sera from patients with SSc (n=3) and healthy controls (HCs) (n=3) were subjected to the detection of global antibodies (online supplemental table S1). By applying the DEEP SEQ proteomics platform, we identified 4798 proteins in total (≤1% false discovery rate (FDR) at peptide level). Among these, 238 enriched proteins were significantly upregulated in SSc in contrast to HCs in IP-MS analysis targeting putative autoantibodies against antigen pools (figure 2B,C). Not all of the 238 identified proteins serve as targets for autoantibodies; some may exhibit non-specific binding due to the limitations of quantitative proteomics techniques. Therefore, further verification is necessary to screen for potential autoantibody candidates within this group of proteins. To confirm the validity of antigen capture process, topoisomerase I (Topo I) was detected by western blotting following IP in a patient who was positive for anti-Topo I antibody (ATA), whereas undetectable in an ATA-negative patient (online supplemental figure S1A,B). In addition, protein-protein interaction networks showed the emergence of protein interaction ‘hot spots’ surrounding Topo I, one of the well-defined antibody targets and frequently associated with SSc^23^ (figure 2D). Among Topo I-associated proteomes, PRMT1, PRMT5, HK-1 and CD5L were over-represented in patients with SSc than HC (figure 2E). Since literature results suggested PRMT1, PRMT5, HK-1 and CD5L with promising pathophysiological relevance,^24–28^ we then focused our investigations on these four proteins as potential autoantibody targets.

10.1136/ard-2024-225596.supp13 Supplementary data

10.1136/ard-2024-225596.supp1 Supplementary data

### Validation of anti-PRMT5 antibody as a specific autoantibody for SSc

**Figure 3:**
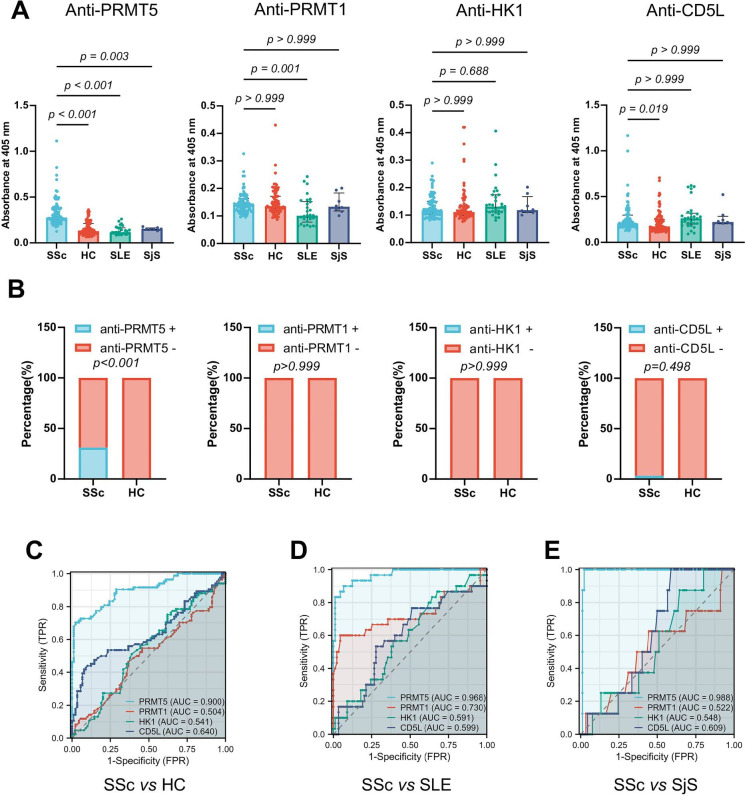
Validation of anti-PRMT5 antibody as a specific autoantibody for SSc. (A) Antibodies against PRMT1, PRMT5, HK-1 and CD5L, determined by ELISA, in serum of 90 patients with systemic sclerosis (SSc), 30 patients with systemic lupus erythematosus (SLE), 8 patients with Sjögren’s syndrome (SjS) and 84 healthy controls (HCs). Data of A are presented as median±IQR, each dot representing one sample. P values were determined by Kruskal-Wallis test with Dunn’s multiple post hoc tests. P values are indicated in the figures. (B) Bar graphs demonstrating the proportion of patients positive or negative for the antibodies against PRMT5, PRMT1, HK-1 and CD5L. The positivity of serum antibody levels was determined if the values were above the 99th percentile as the upper limit of healthy donors. P values were determined by Fisher’s exact test. (C–E) Illustrations of the receiver operating characteristic (ROC) curves, plotted based on the serum levels of antibodies against PRMT5, PRMT1, HK-1 and CD5L in patients with SSc, compared with HC (C), SLE (D) and SjS (E), respectively. The values of the area under the curve (AUC) represent as indicated.

To confirm the discovery results, serum levels of antibodies against PRMT1, PRMT5, HK-1 and CD5L were then measured by ELISA in a primary validation cohort, including 90 patients with SSc, 30 patients with systemic lupus erythematosus (SLE), 8 patients with Sjögren’s syndrome (SjS) and 84 sex-matched and age-matched HC (online supplemental table S2, figure 1B). Serial dilutions for serum were applied to determine the optimal condition for ELISA and calculate values of area under the curve (AUC) (online supplemental figure S2A–D). Serum levels of antibodies against PRMT5, measured as absorbance signals at 405 nm by ELISA, were significantly higher in patients with SSc, compared with HC or the patients with SLE and SjS (SSc vs HC p<0.001; SSc vs SLE p<0.001; SSc vs SjS p=0.003) (figure 3A). Consistently, when calculated by the values of AUC using serial dilutions, serum levels of anti-PRMT5 antibodies also demonstrated an increase in patients with SSc relative to HC, SLE or SjS (online supplemental figure S3A). On the contrary, the level of anti-CD5L antibodies showed a moderate increase in patients with SSc relative to HC (p=0.019) but was comparable with the patients of SLE or SjS. No significant difference was found in serum levels of antibodies against PRMT1 or HK1 across SSc, HC, SLE and SjS (figure 3A, online supplemental figure S3B–D). Next, to exclude technical false positivity, non-relevant anti-ZIKV envelope DIII virus antibodies^29^ were tested as undetectable in both patients with SSc and HC (online supplemental figure S4A–C). Furthermore, using the 99th percentile as the upper limit of normal, anti-PRMT5 antibodies were present in 31.11% of patients with SSc (28/90) and absent in HC (0/84) (figure 3B), with sensitivity, specificity, positive predictive value and negative predictive value of 70.24%, 97.78%, 96.72% and 77.88%, respectively. The positivity of anti-PRMT5 antibodies in SSc was greater than anti-PRMT1, HK-1 and CD5L antibodies (figure 3C–E, online supplemental figure S3E–G). Interestingly, anti-PRMT5 antibodies also demonstrated the ability to differentiate SSc from the patients of SLE and SjS with AUC of 0.968 and 0.988, respectively (figure 3D, E). Following this, we proceeded to validate the levels of anti-PRMT5 antibodies in a recently recruited, independent group of patients with SSc. Consistently, we observed significantly elevated levels of anti-PRMT5 in the sera of these patients with SSc compared with the HC (online supplemental table S3, online supplemental figure S5A,B). Furthermore, we validated the elevated levels of anti-PRMT5 antibodies in sera from these patients with SSc and HC determined using microarray (online supplemental figure S5C,D). Overall, these results indicate that the anti-PRMT5 antibody is a specific surrogate biomarker for SSc.

10.1136/ard-2024-225596.supp2 Supplementary data

10.1136/ard-2024-225596.supp3 Supplementary data

10.1136/ard-2024-225596.supp4 Supplementary data

10.1136/ard-2024-225596.supp5 Supplementary data

### Correlations of the serum levels of anti-PRMT5 antibodies with clinical features of SSc

**Figure 4:**
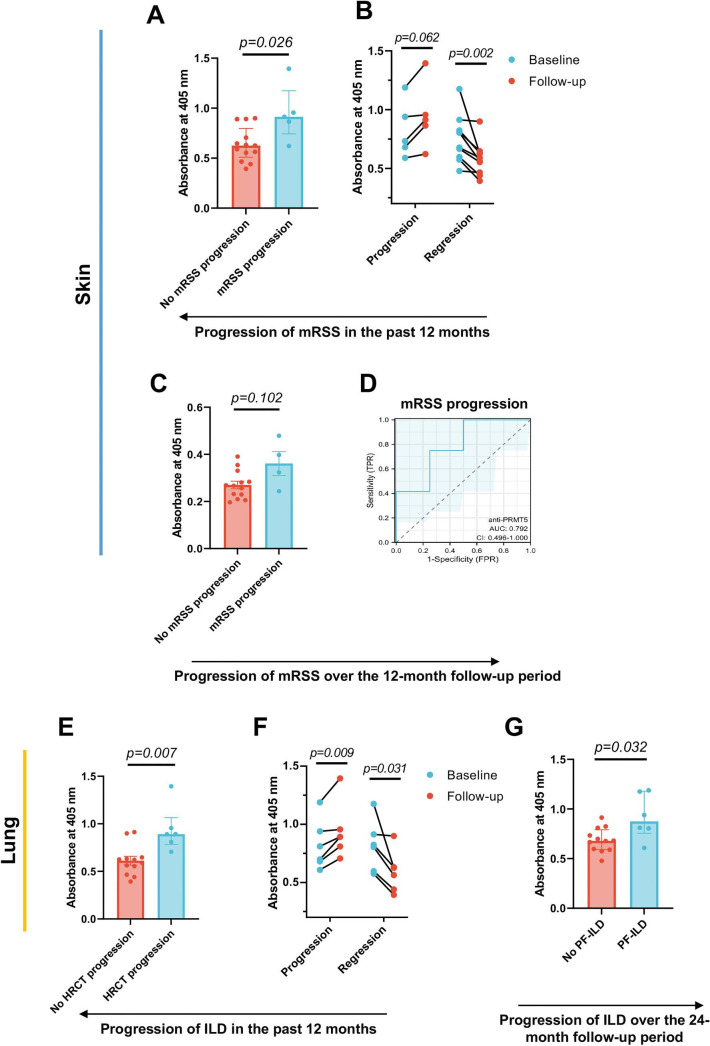
Correlations between anti-PRMT5 antibody levels and the progression of skin and lung fibrosis in patients with systemic sclerosis (SSc). (A) Comparison of anti-PRMT5 antibody levels in patients with SSc, who showed progression or no progression defined by an increase (≥ 25%) in their total mRSS score in the past 12±2 months. (B) Paired comparison analysis of anti-PRMT5 antibody levels of patients with SSc with progression or regression in total mRSS, comparing between the baseline and the follow-up mRSS score with an interval of 12±2 months. (C) Comparison of anti-PRMT5 antibody levels in patients with SSc, with and without skin fibrosis progression, was evaluated prospectively. Skin fibrosis progression was defined as an increase in the modified Rodnan skin score (mRSS) of ≥25% from baseline in the follow-up visit (12 months after baseline). Comparison data are shown as bar graphs with individual values, where each dot represents one sample, and the median and quartiles are indicated. (D) Illustrations of the receiver operating characteristic (ROC) curves, plotted based on the serum levels of antibodies against PRMT5, comparing between patients with SSc with or without mRSS progression prospectively. The values of area under the curve (AUC) represent as indicated. (E) Comparison of anti-PRMT5 antibody levels in patients with SSc-interstitial lung disease (ILD), who showed progression or no progression in their high-resolution CT (HRCT), compared with the HRCT undertaken in the past 12±2 months. (F) Paired comparison analysis of anti-PRMT5 antibody levels of patients with SSc-ILD with progression or regression in lung HRCT, comparing the baseline and the follow-up lung HRCT within an interval of 12±2 months. (G) Comparison of anti-PRMT5 antibody levels in patients with SSc developing progressive fibrosing ILD (PF-ILD) or not in the preceding 24-month follow-up. Comparison data in A, C, E and G are shown as bar graphs with individual values, each dot representing one sample, with the median shown as a continuous line and the quartiles as discontinuous lines. Data in A, C, E and G were analysed by two-sided Mann-Whitney test. Data in B and F were analysed by Wilcoxon matched-pair signed rank test. The p values are indicated in the figures, and p<0.05 was considered statistically significant.

To define the clinical implication of anti-PRMT5 antibodies, we compared the serum antibody levels in patients with SSc with an array of clinical phenotypes (figure 1C). Patients exhibiting progression in skin fibrosis, as determined by a 25% increase in the modified Rodnan skin score (mRSS) compared with the previous visit within a 12-month period, demonstrated elevated serum levels of anti-PRMT5 antibodies (figure 4A). With respect to the relation between anti-PRMT5 antibodies and skin or lung score trajectories of the patients with SSc, we investigated if the occurrence and dynamic changes of these antibodies may fluctuate in parallel with the skin and lung changes during the disease course. Follow-up investigations revealed an elevation in anti-PRMT5 antibodies among patients exhibiting progression in mRSS and a decline in those with mRSS regression (figure 4B). To assess the predictive value of baseline anti-PRMT5 antibody levels for skin fibrosis progression over a prospective 12-month period, we examined patients with SSc manifesting skin fibrosis progression, which was defined as an increase in mRSS ≥25% from baseline in the follow-up visit (12 months after baseline). The patients with SSc with mRSS progression displayed a numerical elevation in baseline levels (p=0.102) of anti-PRMT5 antibody compared with the patients with SSc without skin fibrosis progression (figure 4C). Anti-PRMT5 antibodies also demonstrated the ability to differentiate patients with SSc with mRSS progression from the patients with SSc without mRSS progression with an AUC of 0.792 (figure 4D). To test the potential of anti-PRMT5 antibody as a candidate disease indicator, we took a thorough follow-up for patients with SSc, assessing skin mRSS score and examining anti-PRMT5 levels every 3 months. Remarkably, we observed a parallel change between anti-PRMT5 levels and mRSS scores (online supplemental figure S6A,B).

10.1136/ard-2024-225596.supp6 Supplementary data

Likewise, patients having experienced progression in lung fibrosis, determined by an increased involvement of semiquantified areas in high-resolution CT (HRCT) in the past 12 months, demonstrated a significant elevation in anti-PRMT5 antibody levels (figure 4E). Similarly, the trends of anti-PRMT5 antibodies exhibited a parallel change in patients with SSc with HRCT progression or regression, as compared with the follow-up HRCT score (figure 4F). Patients with SSc who fulfilled the criteria of progressive fibrosing interstitial lung disease (ILD) (PF-ILD) in the subsequent 24-month follow-up demonstrated significantly increased basal levels of anti-PRMT5 antibodies compared with the patients with SSc without developing PF-ILD (figure 4G).

**Figure 5:**
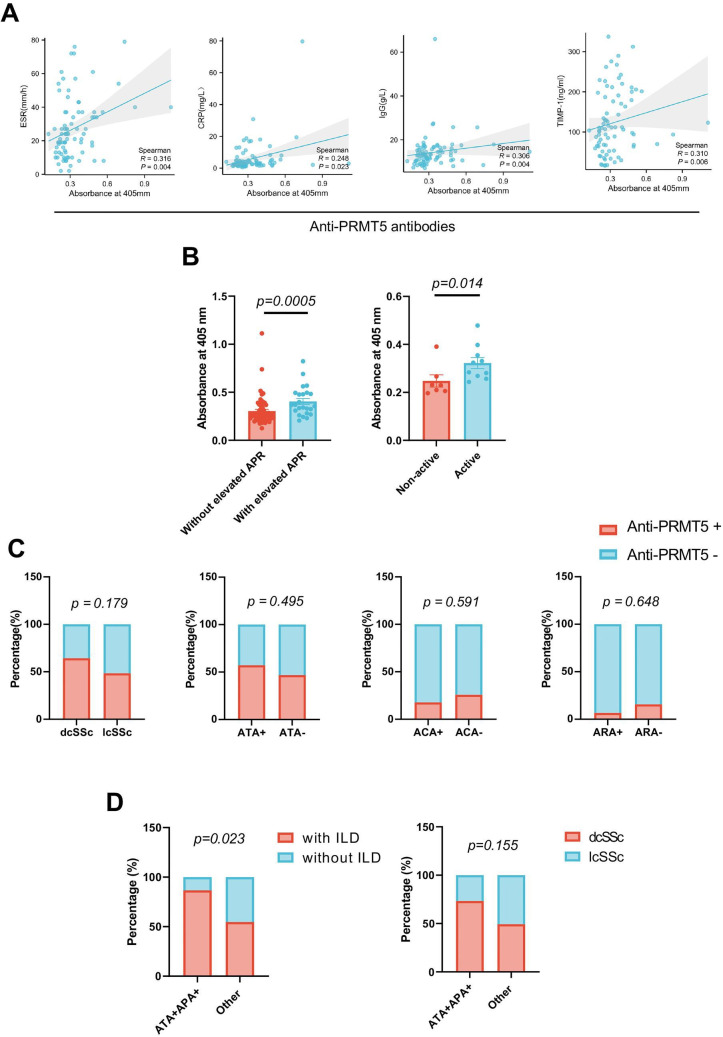
Correlations between anti-PRMT5 antibody levels and inflammatory and autoimmune markers in patients with systemic sclerosis (SSc). (A) Correlations of the serum levels of anti-PRMT5 antibodies with erythrocyte sedimentation rate (ESR), C reactive protein (CRP), IgG and tissue inhibitor of matrix metalloproteinase (TIMP)-1 in SSc. (B) Comparison of anti-PRMT5 antibody levels between patients with SSc with elevated acute phase reactant (APR) levels and the individuals without, stratified as having at least one of the following or not: C reactive protein ≥6 mg/L, ESR ≥28 mm per hour or platelet count ≥330×10⁹/L (left). Comparison of anti-PRMT5 antibody levels between active patients with SSc and non-active patients with SSc, stratified according to the criteria for active disease defined in focuSSced^30^ study (right). (C) Comparison of the positivity of anti-PRMT5 antibodies in patients with SSc with different clinical subsets, including diffuse cutaneous SSc (dcSSc) versus limited cutaneous SSc (lcSSc), positive versus negative for anti-topoisomerase I antibody (ATA), anticentromere antibody (ACA) and anti-RNA polymerase III antibody (ARA). (D) Comparison of percentages of interstitial lung disease (ILD) between patients with SSc double positive for both ATA and anti-PRMT5 antibodies (APA) or not (left). Comparison of percentages of dcSSc versus lcSSc between patients with SSc double positive for both ATA and APA or not (right). Data in A were analysed using non-parametric Spearman correlation analysis. Data in B were analysed by two-sided Mann-Whitney test. Contingency data in C and D were analysed using Fisher’s exact test. The p values are indicated in the figures, and p<0.05 was considered statistically significant.

Furthermore, serum anti-PRMT5 antibodies correlated positively with the levels of acute phase reactants (APRs) like erythrocyte sedimentation rate (ESR) and C reactive protein (CRP), as well as IgG and tissue inhibitor of metal protease 1 (TIMP-1) in SSc (figure 5A). Furthermore, patients with SSc with elevated APR levels, defined as having at least one of the following, CRP ≥6 mg/L, ESR ≥28 mm per hour or platelet count ≥330×10⁹/L, showed higher serum levels of anti-PRMT5, compared with the patients with SSc without APR elevation (figure 5B). According to the criteria for active disease defined in focuSSced^30^ study, we found that active patients with SSc also displayed a greater abundance of anti-PRMT5 antibody (figure 5B). Moreover, we found positive correlations between anti-PRMT5 antibody levels and concentrations of IL-6, tumour necrosis factor alpha, IL-10 and IL-8 in patients with SSc (online supplemental figure S6C). Together, our data may indicate a potential link between anti-PRMT5 antibody and the inflammatory status of patients with SSc.

Patients with SSc with diffuse cutaneous involvement (dcSSc) demonstrated a relatively higher positivity for anti-PRMT5 antibody compared with the patients with SSc with limited cutaneous involvement (lcSSc) but without statistical significance (dcSSc vs lcSSc: 56.34% vs 43.66%, p=0.179, figure 5C). Additionally, no relevance was found between anti-PRMT5 antibody and other known SSc-specific antibodies, including ATA, ACA and ARA (figure 5C). Interestingly, 15 out of 90 (16.67%) patients were double positive for ATA and anti-PRMT5 antibodies. Notably, within this group, 13 out of 15 (86.67%) patients manifested evidence of ILD on HRCT, a higher proportion compared with the non-double positive patients (41/75, 54.67%), with statistical significance (p=0.023, figure 5D). Interestingly, among 18 follow-up patients, all 3 individuals who tested positive for both ATA and anti-PRMT5 antibody experienced ILD progression and fulfilled the criteria of PF-ILD within the preceding 24-month follow-up. Additionally, patients with SSc who are double positive for ATA and anti-PRMT5 antibodies also exhibited a numerical predominance for the diffuse cutaneous subset (11/15, 73.33%) compared with the limited cutaneous subset (4/15, 26.67%) but without statistical significance (figure 5D). No correlations were found between serum levels of anti-PRMT5 antibodies with other clinical parameters in terms of age, sex, disease duration, therapeutic backgrounds, commodity diseases, current mRSS, the presence of digital ulcer (DU), pulmonary arterial hypertension (PAH), telangiectasia or the pattern of nailfold capillaroscopy (data not shown). The data suggest that anti-PRMT5 antibodies are more closely associated with the disease trajectory observed in the skin and lungs of patients with SSc, surpassing the correlation with their current level of involvement.

Furthermore, PRMT5 was more pronounced in fibroblasts and moderately increased in endothelial cells in the dermis of patients with SSc relative to HC (online supplemental figure S7A–C). As the apoptosis of endothelial cells contributes to the pathogenesis of SSc as one of the initial steps,^31 32^ we also observed the significantly increased cell counts of apoptotic PRMT5-positive endothelial cells in the dermis of patients with SSc (online supplemental figure S8), indicating the potential underlying mechanism that PRMT5 may be exposed from apoptotic endothelial cells, triggering autoimmune response subsequently. PRMT5 was observed to be expressed in CD3^+^ T cells and CD68^+^ macrophages of skin, however, without statistical difference between patients with SSc and HC (data not shown).

10.1136/ard-2024-225596.supp7 Supplementary data

10.1136/ard-2024-225596.supp8 Supplementary data

### Induction of skin and lung fibrosis by immunisation with PRMT5

**Figure 6:**
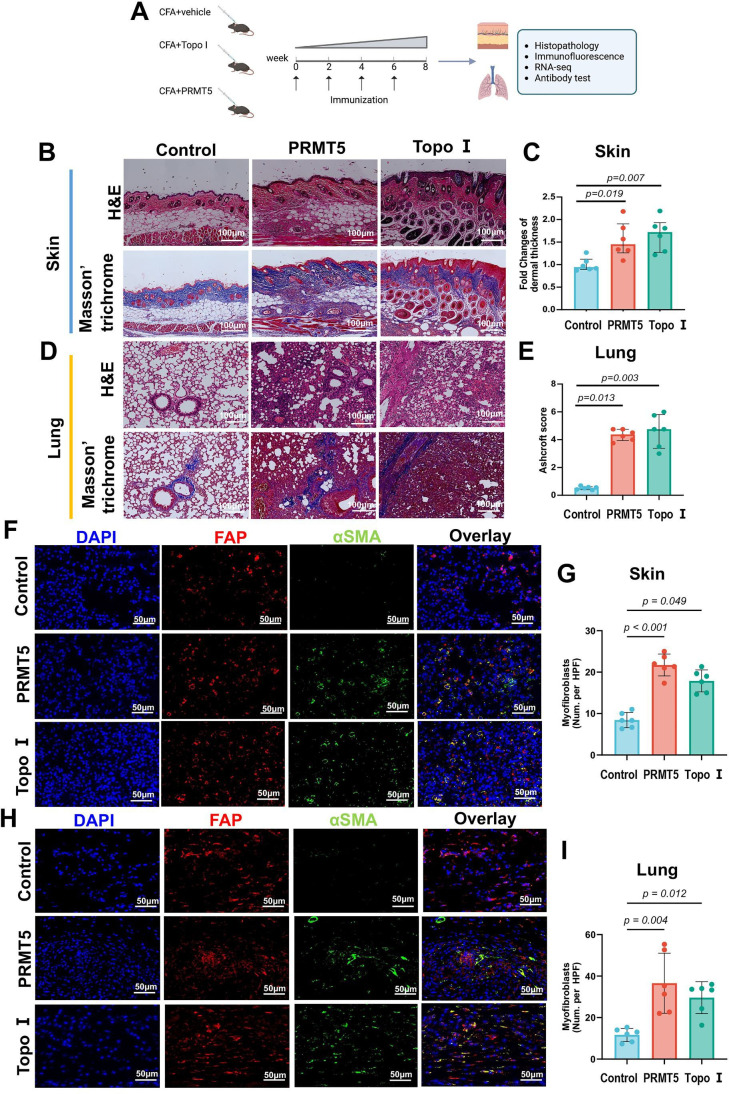
Induction of skin and lung fibrosis in mice immunised by recombinant protein PRMT5. (A) Immunisation with recombinant protein PRMT5 or DNA topoisomerase I (Topo I), along with complete Freund’s adjuvant (CFA) four times subcutaneously with an interval of 2 weeks. Skin and lung tissue samples were collected and followed by the pathological examination (n=6 independent biological samples per group). (B) Representative H&E and Masson’s trichrome staining of the skin shown at 200-fold magnification (scale bars=100 µm). (C) Quantification of dermal thickness, which are normalised to controls. (D) Representative H&E and Masson’s trichrome staining of the lungs shown at 200-fold magnification (scale bars=100 µm). (E) Ashcroft scores were assessed and normalised to controls. (F, H) Representative immunofluorescence staining for αSMA (green) and costaining with FAP (red) in the dermis (F) or lungs (H) of mice treated with PRMT5, or Topo I, along with CFA, at 400-fold magnification (F and H; scale bars=50 µm). (G, I) Numbers of αSMA-positive fibroblasts per high power field (HPF) in the skin (G) and lungs (I) are quantified. All data are presented as median±IQR, each dot representing one sample. P values were determined by Kruskal-Wallis test with Dunn’s multiple post hoc test (C, E, G and I). P values are indicated in the figures.

To elucidate the contribution of anti-PRMT5 antibodies to the development of SSc, we immunised mice with recombinant protein PRMT5 (figure 6A). Skin fibrosis and lung fibrosis were examined histopathologically 8 weeks after initiation of PRMT5 treatment. Treatment with PRMT5/complete Freund’s adjuvant (CFA), in contrast to the treatment with vehicle (Veh)/CFA, resulted in skin fibrosis with increased dermal thickness. In addition, there was no significant difference between the mice treated with PRMT5 and Topo I (figure 6B,C). Similarly, ILD was observed in PRMT5/CFA-treated mice, exhibiting extensive inflammatory infiltration and diffuse fibrosis, with remarkably increased Ashcroft score than Veh/CFA-treated control mice (figure 6D,E). In addition, the Ashcroft score was comparable between the mice treated with PRMT5 and Topo I (figure 6D,E). Immunofluorescence costaining showed an increased number of α-smooth muscle actin (αSMA^+^) fibroblast activation protein (FAP^+^) myofibroblasts in the skin and lungs of mice treated with PRMT5/CFA, compared with the control mice treated with Veh/CFA (figure 6F–I).

To further investigate the serological antibody response, we examined the induction of anti-PRMT5 or Topo I antibodies in sera of mice immunised with recombinant PRMT5 or Topo I, emulsified in CFA, through ELISA. Our findings revealed higher levels of anti-PRMT5 antibodies in sera of mice immunised with recombinant PRMT5, while higher levels of anti-Topo I antibodies in sera of mice immunised with recombinant Topo I (online supplemental figure S9). Further multiplexed immunofluorescence staining was performed by labelling with antibodies against CD45, CD3, CD68 and CD20, which have been reported as markers for pronounced infiltrating immune cell types in SSc.^1 3^ We found a remarkable increase in immune cell infiltration in the skin of PRMT5 immunised mice, including T cells, macrophages and B cells (online supplemental figure S10A,B). Immune infiltration dominant by T cells and macrophages was also found in the lungs of the mice immunised by PRMT5 plus CFA, compared with the Veh /CFA-treated control mice (online supplemental figure S10C,D).

10.1136/ard-2024-225596.supp9 Supplementary data

10.1136/ard-2024-225596.supp10 Supplementary data

To further explore the impact of anti-PRMT5 antibodies on mice and unravel underlying mechanism, we conducted RNA-seq analysis on skin and lung tissues obtained from the mice immunised with PRMT5/CFA or Veh/CFA. Applying a threshold of p<0.05 and |log2 fold change (FC)| ≥1, we identified a total of 4205 and 1169 differentially expressed genes (DEGs) in the skin and lungs, respectively (in the skin, 2681 upregulated DEGs and 1524 downregulated DEGs; in the lungs: 640 upregulated DEGs and 529 downregulated DEGs), comparing between PRMT5/CFA-treated mice versus Veh/CFA-treated control mice (online supplemental figure S11A,B,E,F). The gene sets encompass numerous genes previously implicated in the pathogenesis of SSc, such as *Acta2*, *Col1a1*, *Col3a1*, *Smad3*, *Ctgf*, *Msr1*, *Cd4*, *Cd8a* and *Cd68*, supporting the potential of anti-PRMT5 in induction of SSc-like skin changes. Furthermore, the upregulated gene set identified in the lungs of PRMT5/CFA immunised mice included the overlapping DEGs observed in the skin, such as *Smad3*, *Msr1* and *Cd8a*, along with other fibrosis-associated genes like *Cd163*, *Shh*, *Edn1* and *Fgf12* (online supplemental figure S11E,F).

10.1136/ard-2024-225596.supp11 Supplementary data

To elucidate the functional associations of the identified gene signatures, we performed a gene enrichment analysis for Gene Ontology (GO). This analysis revealed the enrichment of genes involved in immune response and extracellular matrix organisation as key biological processes in the skin of PRMT5/CFA-immunised mice, suggestive of the activation of the immune system and fibrosis process that mirrors characteristics observed in patients with SSc (online supplemental figure S11C). Consistent with this notion, we observed the upregulation of the signalling pathways previously associated with SSc,^3 33^ such as interleukin (IL)-6, IL-4, IL-17, IL-1β, toll-like receptor, vascular endothelial growth factor (VEGF) signalling, JAK-STAT signalling as well as pathways involved in wound healing and extracellular matrix organisation (online supplemental figure S11C). Further analysis using Reactome database demonstrated changes in VEGF or PDGF-mediated signalling, antigen presentation, extracellular matrix organisation, assembly of collagen fibrils as well as cell surface interaction at the vascular wall (online supplemental figure S11D).

In addition, systemic analysis using GO and Reactome databases demonstrated several terms relevant to SSc in the lungs of PRMT5/CFA-immunised mice, including ‘response to IL-13’, ‘wound healing involved in inflammatory response’, ‘response to hypoxia’, ‘antigen receptor-mediated signalling pathway’, ‘classical antibody-mediated complement activation’ and ‘immunoregulatory interactions between a lymphoid and a non-lymphoid cell’ (online supplemental figure S11G,H).

We then delved into signalling pathways governed by DEGs via further Ingenuity Pathway Analysis (IPA). Alongside the activation of numerous fundamental immune and inflammatory responses, as well as cytokine signalling in skin tissues from the mice immunised with PRMT5 (online supplemental figure S12A), our analysis pinpointed the upregulation of a cascade of fibrosis-associated signalling pathways integral to the pathogenesis process,^4 33^ including STAT3, PDGF and G-proteincoupled receptor (online supplemental figure S12). Signalling pathways associated with tissue and matrix regulation have been over-represented in the skin tissues of PRMT5 immunised mice, such as ‘wound healing signalling pathway’, ‘extracellular matrix organisation’ and ‘collagen biosynthesis and modifying enzymes’. Conversely, the suppression signal like ‘inhibition of matrix metalloproteases’ was significantly inhibited (online supplemental figure S12A). This comprehensive understanding is summarised through a graphical network encompassing canonical pathways, upstream regulators and biological functions (online supplemental figure S12B).

10.1136/ard-2024-225596.supp12 Supplementary data

Therefore, immunisation with PRMT5 provokes SSc-mimicking inflammation and fibrosis in the skin and lungs in vivo. These findings may indicate PRMT5 as a potential contributing antibody target in SSc, likely participating in the processes of autoimmune response and myofibroblast activation.

## Discussion

Our study here is the first to identify PRMT5 as a novel autoantibody target of the autoimmune response in patients with SSc based on large-scale proteomics with automated DEEP SEQ.^22^ Anti-PRMT5 antibodies are present in 31.11% of patients with SSc and absent in HCs. We found the dynamic changes of anti-PRMT5 antibodies in parallel with the progression or regression of skin fibrosis and ILD of SSc; monitoring the levels of anti-PRMT5 antibodies may therefore enable early detection and the initiation of early intervention for the patients with SSc with a higher risk of mRSS progression and development to PF-ILD during follow-up. Histopathological evaluations further revealed an overexpression of PRMT5, predominantly localised within fibroblasts and endothelial cells, among patients with SSc. Furthermore, mice immunised with PRMT5 showed marked tissue fibrosis coupled with immune cell infiltration within both dermal and pulmonary tissues, as well as serological antibody response, mirroring the pathognomonic changes typically associated with human SSc. RNA-seq demonstrated immunisation with PRMT5-induced multiple profibrotic and proinflammatory transcriptional networks in mice. Collectively, these data thus indicate the potential role of anti-PRMT5 as a contributing autoantibody in the development of SSc.

The first step of the current study is a screening for autoantigens with an exploratory strategy using high-throughput proteomics. Lysates of the cell lines were used as a source of putative autoantigens. The antibody-antigen complex was enriched via IP and analysed with large-scale proteomics based on automated DEEP SEQ MS as we established previously.^22^ In contrast to the DNA sequencing platforms, the protein-level array poses challenges attributed to the wide dynamic span in protein expressions and vast diversity in post-translational modifications, coupled with the lack of an amplification strategy analogous to PCR, limiting genome-wide protein characterisation, particularly for signal transduction and other key regulatory factors that are often present in low abundance. To address this issue, we employed an established genome-scale proteome quantification by DEEP SEQ MS, which is based on simple detergent lysis and single-enzyme digest, extreme, orthogonal separation of peptides and true nanoflow liquid chromatography (LC)-MS/MS, significantly increasing the scale of proteome coverage.^22^ In fact, omitting protein crosslinking may avoid the complexities associated with diverse protein conformations. Subsequent studies in patient cohort demonstrated that the autoantibodies against PRMT5 can be detected in SSc with high diagnostic performance. In this case, these results support the feasibility of this coordinated workflow combining thorough screen and functional validation for the identification of optimal diagnostic and therapeutic antibody candidates in general.

The presence of autoantibodies in patients does not mean that the autoantibodies can mediate the clinical manifestations. Therefore, the challenge is to clarify the role of autoreactivity in a clinical scenario and determine whether autoreactivity is crucial or merely incidental. The validity of anti-PRMT5 antibodies rather than anti-PRMT1, HK-1 or CD5L antibodies with higher specificity and sensitivity for SSc leads to the next in-depth investigation for anti-PRMT5 in SSc. Besides, anti-PRMT5 antibodies demonstrated promising predictive value for the progression of skin and lung disease in SSc. Therefore, the role of autoimmunity of anti-PRMT5 was then assessed in an immunisation model, which has been extensively used for model establishment, such as experimental allergic encephalomyelitis,^34^ collagen-induced arthritis,^35^ glucose-6-phosphate isomerase-induced arthritis^36^ as well as Topo I immunised SSc.^23^ Indeed, induction of tissue fibrosis and immune infiltration in mice immunised by recombinant protein PRMT5 illustrated that the antibody response to PRMT5 was likely to result in SSc-like manifestations. As anticipated, we observed several upregulated DEGs in he skin related to T cell, B cell and macrophage response, as well as fibroblast activation. Through GO and Reactome analysis, we revealed characteristic changes in both the skin and lungs of PRMT5-immunised mice, clearly distinguishing them from controls. These changes included several key functional categories: (1) we observed various biological processes in PRMT5-immunised mice related to autoimmune responses, with particular emphasis on processes linked to antigen presentation and immune cell activation; (2) we showed the increased production of profibrotic cytokines, including IL-4, IL-13, IL-6 and IL-1β; (3) we found multiple biological processes related to fibroblast activation and fibrotic tissue remodelling, including extracellular matrix organisation, biosynthesis and assembly; and (4) we observed the activity of several key profibrotic signalling, such as VEGF, PDGF, JAK-STAT and Toll-like receptor-mediated signalling. Further comprehensive analysis encompassing canonical pathways, upstream regulators and biological functions was also conducted by IPA. Thus, our unbiased RNA-seq analysis highlights immune response and tissue remodelling as a characteristic feature across the skin and lungs in the mice immunised with PRMT5, mirroring aspects of human SSc pathology.

The overexpression of PRMT5 in the fibroblasts and endothelial cells of SSc has provided the rationale that PRMT5 might be related to the fibroblast activation and endothelial dysfunction, which have been revealed as crucial in the pathogenesis of SSc.^2 4^ PRMTs have been shown to play critical roles in disease through methylation of arginine residues on histone or non-histone proteins. Of note, circulating monomethyl arginine and asymmetrically dimethylated arginine can inhibit the function of nitric oxide (NO) synthase, which generates NO. Interestingly, attenuated NO bioavailability results in a milieu of inflammation and oxidative stress in SSc, leading to vasculopathy and subsequent fibrosis and reshaping of NO metabolism has been proven to be an effective treatment of SSc-associated vasculopathy, especially for DU and PAH.^37 38^ PRMT5 was identified as a symmetrical dimethyltransferase ubiquitously expressed in the kidneys, skin, lungs and other tissues.^39^ PRMT5 inhibitors have demonstrated efficiency in treating mouse models of acute graft-versus-host disease, as elucidated by prolonged survival and ameliorated disease severity, along with decreased T cell proliferation and cytokine production.^40^ PRMT5 has been reported to regulate T cells through various pathways, including promoting retinoid-related orphan receptor (ROR)-γt activity and adjusting the Klf2-S1pr1 pathway.^41 42^ Arginine methylation mediated by PRMTs has emerged as a critical mechanism implicated in fibrosis.^43–47^ Notably, fibroblast-specific deletion of PRMT5 significantly reduced pressure overload-induced cardiac fibrosis. PRMT5 has been shown to regulate transforming growth factor beta (TGF-β)/Smad3-dependent fibrotic gene transcription, potentially through histone methylation crosstalk, and plays a critical role in cardiac fibrosis and dysfunction.^45^ Similarly, the contribution of PRMT5 to fibrosis has been confirmed in an Adriamycin-induced cardiac fibrosis model.^46^ These findings suggest that PRMT5 may serve as a critical mediator in regulating TGF-β-stimulated fibroblast activation and tissue fibrosis. Since protein methylation is a targetable modification and advanced drug development of PRMT5 inhibitors has been achieved, the therapeutic potential of targeting PRMT5 appears promising.

In sum, this study has identified anti-PRMT5 antibodies as a novel biomarker for SSc. The current data suggest the potential underlying mechanism driven by PRMT5 as a target of autoimmunity and consequently resulting fibrosis in SSc. However, the exact role of anti-PRMT5 in SSc needs further elucidation.

## Method

A detailed description of all materials and methods is provided in online supplemental material.

All human studies were approved by the ethical committee of the Medical Faculty of Fudan University. All patients and controls signed an informed consent form approved by the local institutional review board.

All animal experiments were carried out in strict accordance with international and local guidelines for animal care and use. Mice were maintained under pathogen-free conditions, with a standard diet, water ad libitum and 12 hour light/12 hour dark cycle. Mice were 6-week old at the start of experiments, and up to six mice were housed in one cage.

Statistical measures, including the number of samples, descriptive statistics (median and IQR) and significance, are reported in the figures and figure legends. P<0.05 was considered statistically significant.

## Data Availability

Data are available upon reasonable request. All data relevant to the study are presented in the article or uploaded as supplementary information.
