# Supplementary material for: Identification and validation of anti-protein arginine methyltransferase 5 (PRMT5) antibody as a novel biomarker for systemic sclerosis (SSc)
Source: Ann Rheum Dis. 2024 Apr 29;83(9):1144–55. doi: 10.1136/ard-2024-225596 (PMC11420721; doi:10.1136/ard-2024-225596)
Supplement: Supplementary data [file ard-2024-225596supp013.pdf]

Supplemental Material

Supplemental Methods

Figure S1-S12

Table S1-S4

Supplemental Methods

Study design

The study aims to identify and validate novel autoantibodies in systemic sclerosis (SSc) and further elucidate the contribution of autoantibody to the pathogenesis of SSc (Figure 1). We develop a global autoantibody profiling strategy that integrates immunoprecipitation (IP) coupled with ultrasensitive quantitative proteomics (Figure 2A). In brief, serum was obtained from the SSc patients or healthy donors, then incubated with the antigen pool which was obtained from cell lysates of 4 different cell lines. The corresponding cell types have previously implicated in the pathogenesis of SSc. Antigens bound by antibodies were enriched through immunoprecipitation, subjected to on-beads digestion, and subsequently processed using an automated deep efficient peptide sequencing and quantification (DEEP SEQ) mass spectrometry platform [1]. Subsequently, we validated the diagnostic accuracy of identified autoantibodies in an SSc cohort. The levels of anti-PRMT5 antibodies were correlated with clinical characteristics and disease outcomes. The expression of PRMT5 in the skin of SSc patients was also confirmed with immunofluorescence staining. In order to reveal the potential contribution of anti-PRMT5 antibodies to the development of SSc, inflammation and fibrosis in both skin and lungs were examined using multiplexed immunofluorescence staining and RNA-seq in mice immunized with PRMT5 and complete Freund’s adjuvant (CFA). The serological antibody response was evaluated using ELISA.

Study Subjects

Liang M, *et al. Ann Rheum Dis* 2024; 83:1144–1155. doi: 10.1136/ard-2024-225596

BMJ Publishing Group Limited (BMJ) disclaims all liability and responsibility arising from any reliance placed on this supplemental material which has been supplied by the author(s)

Serum samples were obtained with informed consent from 90 SSc patients, 30 Systemic lupus erythematosus (SLE) patients, 8 Sjögren's syndrome (SjS) patients and 84 healthy donors. All SSc patients fulfilled the American College of Rheumatology (ACR) / European Alliance of Associations for Rheumatology (EULAR) 2013 criteria [2]. The study was approved by the local ethics committee and informed consent was obtained from all participants.

SSc patients were stratified into diffuse cutaneous SSc (dcSSc) and limited cutaneous SSc (lcSSc) according to the extent of skin involvement [3]. Disease duration was calculated from the onset of the first non-Raynaud's phenomenon (RP) symptom. Modified Rodnan skin score (mRSS), a semi-quantification of the extent and severity of skin involvement in SSc, was performed to assess skin fibrosis based on palpation at 17 anatomical body sites. Interstitial lung disease (ILD) was identified by high-resolution computed tomographic scan (HRCT), that showed fibrosis affecting at least 10% of the lungs, as confirmed by an expert radiologist and a rheumatologist independently. A visual semiquantitative HRCT score was applied. Briefly, the overall HRCT score was graded according to the types and the extent of parenchymal abnormalities in six lung zones as described [4]. Progressive fibrosing ILD (PF-ILD) was diagnosed if fulfilled any of the criteria: a) Fibrosis exceeding 10% of lung volume on recent chest HRCT; b) Forced vital capacity (FVC) decline ≥10% of the normal predicted values within the preceding 24 months; c) FVC decline by 5%-10% of the normal predicted values within the preceding 24 months and with either worsening symptoms or worsening CT scan [5]. SSc patients with elevated acute phase reactant (APR) levels was defined as having at least one of the following or not: C-reactive protein ≥ 6 mg/L, ESR ≥ 28 mm/h, or platelet count ≥ 330 × 10^9/L. Active SSc patients and non-active SSc patients was stratified according to the criteria for active disease defined in focuSSced [6] study. Serum levels of antibodies were measured and clinical evaluations (including mRSS assessment and HRCT scan) were conducted in all the enrolled patients at baseline. Eighteen SSc patients underwent a follow-up serological testing and clinical evaluation during treatment with immunosuppressants and/or anti-

fibrotic drugs. The serum was separated and stored at −80°C. The mRSS assessment and HRCT scan were performed within 3 days of the collection of blood samples. All skin biopsies of SSc patients were taken on the forearm 15 ± 2 cm proximal from the styloid process of the ulna using disposable biopsy punches (#48301, pfm medical, Gifu, Japan).

Quantitative proteomics analysis

The detailed methods can be found in our previous publication [1,7]. Briefly, proteins were digested by 50 mM Trypsin (#PR-V5113, Promega, Madison, WI, USA). Peptides were labelled with 4-plex iTRAQ reagents (#PN4374321, Sciex, Framingham, MA, USA) and mixed and then loaded to an online three-dimensional chromatography platform for in-depth proteomic quantification. Multiplexed iTRAQ-based quantitative proteomic analysis was performed as described previously [1 7].

Construction of an antigen library

To construct an antigen pool, Jurkat T cells, human dermal fibroblasts (HDFs), THP-1 monocytes and human umbilical vein endothelial cells (HUVECs) were lysed in RIPA buffer consisting of 50 mM Tris-HCl (pH 7.5), 150 mM NaCl, 1 mM EDTA, 10% glycerol, 0.5% sodium deoxycholate, 0.1% SDS, 1 mM PMSF, 1 mM NaF, 1 mM Na_3VO_4 and 1% protease inhibitor mixture (#P8340, Merck, Darmstadt, Germany) at 4°C for 30 min. The concentration of protein was adjusted to 2 mg/ml and mixed with the same volume.

Immunoprecipitation for the extraction of antibody-targeted proteins

The cell lysates were incubated with an equal amount of serum from SSc patients or healthy donors at 4°C for 4 h followed by incubation with Protein A/G agarose (#20422, Thermo Scientific, Waltham, MA, USA) for another 1 h at 4°C. The agarose beads were washed with RIPA buffer followed by elution for further SDS-PAGE or iTRAQ detection.

On-beads digestion and iTRAQ labeling

The beads and antigen-antibody complex were washed 5 times with IP binding buffer (20 mM Tris-HCl, 1 mM EDTA, 150 mM NaCl, 10% glycerol, pH 7.5). The beads were resuspended in 500 µl of 0.5 M Tris (pH 8.5) and incubated with 20 mM TCEP buffer. The antigen-antibody binding to the beads was incubated with 20 µg trypsin at 37°C overnight for in-solution digestion to minimize sample loss. The beads were centrifuged at 12,000 × g for 2 min at room temperature, and flow-through containing peptides was collected afterwards.

Digested peptides were dried by vacuum centrifugation. and reconstituted in 200 µl of 0.1% trifluoroacetic acid and loaded onto SepPak C18 reversed phase plates (#WAT036945, Waters, Milford, MA, USA) to remove salts. The desalted peptides were then eluted with 50 µl of 70% acetonitrile, labeled with multiplexed isobaric tags using 4-plex iTRAQ reagents and mixed and loaded into a liquid chromatography.

Multi-dimensional separation and data acquisition

The three-dimensional online chromatography at the nanoscale is made up of a reversed phase column (200 µm I.D. capillary packed with 20 cm of 5 µm diameter. XBridge C18 resin) as the first dimension, a SAX column (200 µm I.D. 20 cm of 10 µm dia. POROS10HQ resin) as the second dimension and a reversed phase column (25 µm I.D. 120 cm of 5 µm dia. Monitor C18, integrated 1 µm dia. emitter tip) as the third dimension. The final reversed phase column ran at 2-3 nl/min with a 600 min gradient from 2% buffer A to 50% buffer B (A=0.1% formic acid; B=acetonitrile with 0.1% formic acid). The downstream TripleTOF 5600 (Sciex, Framingham, MA, USA) was set in data-dependent acquisition (DDA) mode. For each MS scan with a scan range of 500-1500 m/z and duration of 700 ms, only the top 50 precursors with charge state ranging from +2 to +4 and occurring more than 60 times were selected for MS/MS, which had a maximum time of 230 ms and scan range of 100-1400 m/z. The electrospray voltage was 2.3 kV.

Liang M, *et al. Ann Rheum Dis* 2024; 83:1144–1155. doi: 10.1136/ard-2024-225596

Data processing and protein identification

The mass spectrometry data were subjected to search against the SwissProt database (downloaded on 24 Nov 2021) utilizing the search parameter 4-plex iTRAQ labeling with ProteinPilot V.4.5 (Sciex, Framingham, MA, USA). Peptides were filtered using a peptide spectrum match false discovery rate (FDR) lower than 1%. Peptides that can be assigned to multiple genes were eliminated. We then summed the intensity of each iTRAQ reporter ion for the peptides assigned to single gene to generate an intensity for each gene.

Protein quantification

We adopt the proteome quantification methodology from our previous work [8]. Briefly, in the process of protein quantification and differential expression analysis, the following two assumptions were applied. (1) The protein counts of a non-differentially expressed gene in two samples, expressed as a log2 ratio, are normally distributed as N (0, σ2), where 0 represents the mean and σ2 is the variance that exponentially decreases with average signal intensity of each gene. (2) A sliding window of 500 proteins moving along the axis of log10 (intensity) was introduced and the distribution of log2 ratio of genes was considered as a mixture of the normal distribution N (0, σ2) contributed by non-differentially expressed genes and log2 ratio far from 0 contributed by differentially expressed proteins. By introducing a sliding window consisting of 500 proteins along the intensity axis, we analyzed the distribution of log2 ratios of proteins as a mixture of normal distribution N (0, σ2) from non-differentially expressed proteins and log2 ratios distant from 0 contributed by differentially expressed proteins as previously expressed [8]. The ion intensity in iTRAQ mass spectrometry signal was normalized by using the cumulative intensity of high-confidence non-specific proteins to compare independent experiments and individual samples. By fitting an exponential decay function between the average intensity of the proteins and the σ2 of each window, we calculated the σ2 of each gene to evaluate the technical variations and determine the

significance (*P*-value) of its log2 ratio. A protein was categorized as differentially expressed if it met the following criteria in at least two of the five replicates: (1) iTRAQ intensity ≥ 100; (2) iTRAQ ratio ≥ 1.5; and (3) *P*-value ≤ 0.05.

Enzyme-linked immunosorbent assay (ELISA)

96-well half-area microplates (#CLS3690, Corning, Glendale, Arizona, USA) were coated with 50 µl of 2 ng/µl PRMT5, PRMT1, HK-1, CD5L protein, or non-relevant ZIKA virus domain III antigen, followed by overnight incubation at 4°C. Recombinant PRMT5, PRMT1, HK-1, CD5L proteins were purchased from regent providers as indicated in the Table S4, while ZIKA virus domain III was expressed in-house as previously described [9]. After incubation, the plates were washed three times using 200 µl PBS supplemented with 0.1% (v/v) TWEEN-20 (PBST). 3% (w/v) Bovine Serum Albumin (BSA) in PBS (100 µl / well) was added as blocking buffer, followed by 1 h incubation at 37°C. After three washes, human serum samples were prepared for the experiment through serial dilution (5×, 10×, 20×, and 40×) with 3% BSA, followed by 1.5 h incubation at 37°C. The mouse serum samples were diluted at a ratio of 1:10 for the subsequent ELISA. The plates underwent another round of washing. Next, protein-binding autoantibodies were detected by the addition of 50 µl of the monoclonal anti-Fab-HRP antibody (#I5260, Sigma-Aldrich, St. Louis, MO, USA) diluted 5000 times, followed by a 45-min incubation at 37°C. After this step, the plates were washed five times. The enzyme activity was measured by adding the substrate ABTS (Invitrogen, Carlsbad, CA, USA), and the signal was read at 405 nm using a Microplate Spectrophotometer (Biotek, Winooski, VT, USA). GraphPad Prism 8 version 8.3.0 (GraphPad Software, San Diego, CA, USA) was applied to plot the data and calculate the area under curves.

Microarray

The microarray was used for serum profiling as previous report [10]. The concentrations of recombinant PRMT5 protein (#11074-H32B, Sino Biological)

printed on microarray slides were 0.02125 mg/ml, 0.0425 mg/ml, 0.085 mg/ml, and 0.17 mg/ml. The diluted human serum (1:100) was incubated with each subarray overnight at 4 °C. The arrays were washed with PBST and bound antibodies were detected by incubating with Cy3-conjugated goat anti-human IgG (Jackson ImmunoResearch, PA, USA, Cat#109-165-008) for human sera, which were diluted 1: 1000 in PBST and incubated at room temperature for 1 hour. The microarrays were then washed with PBST and dried by centrifugation at room temperature and scanned by LuxScan 10K-A (CapitalBio Corporation, Beijing, China) with the parameters set as 95% laser power/PMT 550 for IgG. The fluorescent intensity data was extracted by GenePix Pro 6.0 software (Molecular Devices, CA, USA).

Cytokine determination by Luminex

Serum concentrations of IL-6, IFN-gamma, TNF-alpha, IL-2, IL-1beta, IL-13, IL-4, IL-8, IL-10, and IL-12p70 were quantified using the Luminex X-MAP system (Luminex, Austin, TX, USA) following the manufacturer's instructions (LX-MultiDTH-10, LabEx Co., Shanghai, China).

Mouse Experiment

Wide-type C57BL/6 mice were purchased from the Chinese Academy of Science (Shanghai, China) and kept in a pathogen-free environment. Experimental procedures were in accordance with the Animal Care and Use Committee at Fudan University, Shanghai, China. Mice were maintained under pathogen-free conditions, with standard diet, water ad libitum and 12-hour light/12-hour dark cycle. Mice were 6-week-old at the start of experiments and up to six mice were housed in one cage. The protocol was approved by Fudan University, Shanghai, China (2021JS HSYY-052; 2022JS HSYY-054; 2023-HSYY-179JZS).

Immunization of mice with recombinant PRMT5 protein

Recombinant human topo I (#TG2005H-RC3, TopoGen, Buena Vista, CO, USA)

was dissolved in saline to the concentration of 500 units/ml and applied as positive controls, following the described procedure [11]. Recombinant human PRMT5 (#11074-H18H, Sino Biological, Beijing, China) was dissolved in saline to the concentration of 0.57 μg/ml, the molecular concentration of which was equal to topo I. The topo I or PRMT5 solution was mixed 1:1 with CFA (#F5881, Sigma-Aldrich, St. Louis, MO, USA). The mice were injected with vehicle and CFA with the same volume as controls. These solutions (300 μl) were injected 4 times subcutaneously at the indicated locations on the shaved back of the mice with a 26-gauge needle at an interval of 2 weeks. At week 8, mice were sacrificed for further pathological and serological investigation.

Histopathology

All skin sections were obtained at the same anatomical location from the paramidline lower back to minimize regional variations in thickness. Sections were stained with hematoxylin and eosin (H&E) and Masson's trichrome staining. Dermal thickness, defined as the thickness of skin from the top of the granular layer to the junction between the dermis and subcutaneous fat, was examined. Five random measurements per section were obtained [12] [13,14]. All the sections were examined independently by 2 investigators (MRL and LBW) in a blinded manner.

Left middle lobe of lungs was excised and processed with morphologic assessment as previously described [12] [15]. The Ashcroft scale for the visualized evaluation of lung fibrosis is the analysis of Masson's trichrome stained sections, which were randomly captured and microscopically photographed with a 100-fold magnification [16]. Briefly, lung fibrosis was graded on a scale of 0 to 8 by examining randomly chosen fields of the left middle lobe. The grading criteria were as follows: Grade 0, normal lung; Grade 1, isolated alveolar septa with gentle fibrotic changes; Grade 2, fibrotic changes of alveolar septa with knot-like formation; Grade 3, contiguous fibrotic walls of alveolar septa; Grade 4, single fibrotic masses; Grade 5, confluent fibrotic masses; Grade 6, large contiguous fibrotic masses; Grade 7, airbubbles; Grade 8, fibrous obliteration.

Liang M, *et al. Ann Rheum Dis* 2024; 83:1144–1155. doi: 10.1136/ard-2024-225596

Tyramide signal amplification (TSA) staining

Formalin-fixed, paraffin-embedded (FFPE) skin or lung sections fixed in 4% paraformaldehyde were stained using a TSA system as described [17]. We used primary antibodies against PRMT5 (Clone: EPR5772, #ab109451, 1:200, Abcam, Cambridge, MA, USA), FAP (polyclonal, #AF3715, 1:100, R&D Systems, Minneapolis, MN, USA), CD31 (Clone: JC/70A, #ab9498, 1:100, Abcam, Cambridge, MA, USA), CD45 (polyclonal, #20103-1-AP, 1:2000, Proteintech, Rosemont, IL, USA), CD3 (Clone: JRMR-42, #PTM-5013, 1:1000, PTM BIO, Hangzhou, Zhejiang, China), CD68 (Clone: E3O7V, #97778S, 1:1000, Cell Signaling Technology, Danvers, MA, USA) and CD20 (Clone: SP32, #ab64088, 1:200, Abcam, Cambridge, MA, USA) as well as antibody against αSMA (Clone: 1A4, #A5228, 1:1000, Sigma-Aldrich, St. Louis, MO, USA). The signal was amplified and detected with Opal reactive fluorophores (#NEL861001KT, Akoya Biosciences). Nuclei were counterstained using DAPI (0.5 µg/ml, Santa Cruz Biotechnology, Dallas, Texas, USA). Images were captured using a digital slide scanner (PANNORAMIC SCAN II, 3DHISTECH, Öv u. 3., Hungary) and data were collected using NIS-Elements BR version 5.20.01 software (Nikon, Badhoevedorp, Netherlands). We generated 5 high-power fields (HPFs) per each sample at a magnification of 400 ×, which were representative for the images within the set. All images were captured and arranged using the same equipment setting. Quantification of indicated cells was conducted using ImageJ / Fiji software (Version 1.53c, https://fiji.sc).

In addition, myofibroblasts were recognized as single spindle-shaped $\alpha SMA^{+}FAP^{+}$ cells. Cell counts per high-power field (HPF) were examined in 5 random fields of fluorescence-stained sections with a 400-fold magnification.

RNA sequencing (RNA-seq)

The skin tissue and left middle lobe of the lungs from the mice immunized with PRMT5 / CFA or vehicle / CFA were extracted (3 biologically independent samples per group). Total RNA was harvested with Trizol according to the manufacturer and the

Liang M, *et al. Ann Rheum Dis* 2024; 83:1144–1155. doi: 10.1136/ard-2024-225596

cDNA was generated and amplified to form the library, which was sequenced on the BGISEQ-500 platform (BGI Genomics, Shenzhen, China).

The raw data were handled by Skewer and data quality was checked by FastQC v0.11.5. Clean reads were aligned to the GRCm39 (RefSeq assembly GCF_000001635.27) using STAR. The expression of the transcript was calculated by Fragments Per Kilobase of exon model per Million mapped reads (FPKM) using Perl. Differentially expressed genes (DEGs) were determined using the MA-plot-based method with Random Sampling (MARS) model in the DEGseq package between mice immunized with PRMT5 / CFA or CFA. Generally, in MARS model, M = log2C1 - log2C2, and A = (log2C1 + log2C2)/2 (C1 and C2 denote the counts of reads mapped to a specific gene obtained from two samples). The thresholds for determining DEGs are p-value < 0.05 and |log2FC| ≥ 1. Then DEGs were chosen for function and signaling pathway enrichment analysis using GO and Reactome databases. The significantly enriched pathways were determined when $p < 0.05$ and at least two affiliated genes were included.

Ingenuity Pathway Analysis

To explore the signaling pathways influenced by DEGs across identified clusters or groups, we utilized Ingenuity Pathway Analysis (IPA, QIAGEN). The dataset, including DEGs with their fold change, p-value, and adjusted p-value, was imported into IPA. Using the Ingenuity Knowledge Base for Core Expression Analysis, we assessed pathway activation/inhibition levels *via* z-scores, indicating alignment with expected expression patterns. The z-scores > 0 suggest activation; < 0 indicates inhibition, with ≥ 2 or ≤ −2 deemed significant. Pathway significance was determined through right-tailed Fisher's exact test. This significance reflects the probability of the association of molecules from our RNA-seq dataset with the canonical pathway reference dataset. Graphical summary was generated to provide an overview of the major biological themes in our IPA core analysis and illustrate how these concepts interrelate. A machine learning algorithm, relying entirely on prior knowledge, was

employed to score inferred relationships between molecules, functions, and pathways. Networks were constructed from the IPA analysis results using a heuristic graph algorithm.

Statistics

Quantitative data are shown either as bar graphs or scatter plots. Data in bar graphs are presented as median ± interquartile range (IQR) with individual data points plotted as dots. Statistical significance was calculated using the established or custom package in R (version 3.5.3). Further statistical analysis on clinical correlation was also performed using GraphPad prism 8 version 8.3.0. Unless stated otherwise, Mann-Whitney U non-parametric testing was used for all two-group comparisons and Kruskal-Wallis test with Dunn's multiple post hoc test was used for the comparisons involving more than two groups. Relationships between continuous variables were assessed using non-parametric Spearman correlation. p-values < 0.05 were considered statistically significant.

Liang M, *et al. Ann Rheum Dis* 2024; 83:1144–1155. doi: 10.1136/ard-2024-225596

Figure legend of supplementary figures

Figure S1

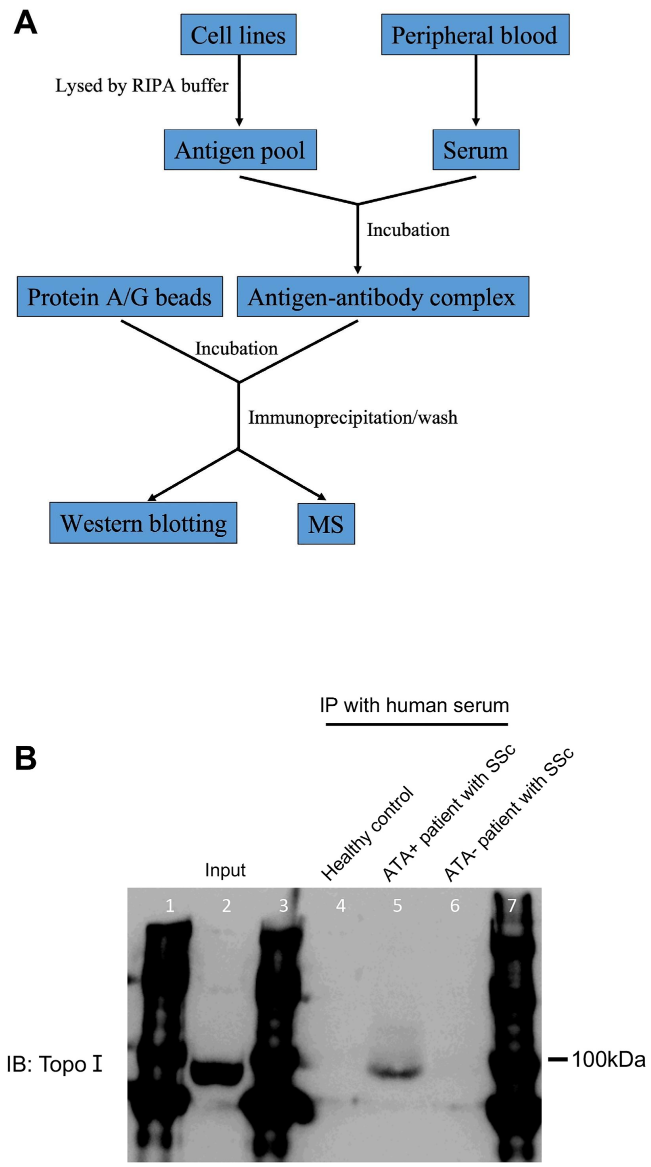

Figure S1. Enrichment process for IgG binding antigens by Immunoprecipitation (IP).

(**A**) Workflow of the experimental set-up for the enrichment of IgG binding antigens. Cell lines of Human Umbilical Vein Endothelial Cells (HUVECs), human dermal fibroblasts (HDFs), Jurkat T cells and THP-1 monocytes were lysed by RIPA buffer,

Liang M, *et al. Ann Rheum Dis* 2024; 83:1144–1155. doi: 10.1136/ard-2024-225596

which were aliquoted as an antigen pool. Peripheral blood was obtained from 3 patients with SSc and 3 healthy donors. The antigen-antibody complex was captured and immobilized by incubation with Protein A/G, followed by enrichment with immunoprecipitation (IP). Lastly, the antigens binding to antibody, which covalently attached to the beads, were eluted for sodium dodecyl sulfate-polyacrylamide gel electrophoresis (SDS-PAGE), followed by western blot to verify the identity of the known antigen, such as DNA topoisomerase I (Topo I). The cell lysate was proceeded with Mass Spectrometry (MS)-based proteomics to identify unknown antigens. (**B**) Verification of enrichment process for antigen-antibody complex by IP. Serum from the SSc patient who is positive or negative for anti-Topo I antibody (ATA) were collected, and incubated with antigen pool as described to form antigen-antibody complex. The antigen-antibody complex was enriched by protein A/G beads and eluted followed by western blotting to verify the identity of the antigen Topo I. Immunoblotting (IB) using the antigen pool without precipitation was performed as an input. The blot for each lane were indicated as follows:

Lane 1, 3, 7: Molecular marker (#PM5100, SMOBIO);

Lane 2: Input;

Lane 4: Pull-down was performed using serum from a healthy control;

Lane 5: Pull-down was performed using serum from an ATA^+ SSc patient (Patient 1 in Table S1);

Lane 6: Pull-down was performed using serum an ATA^- SSc patient (Patient 2 in Table S1)

Figure S2

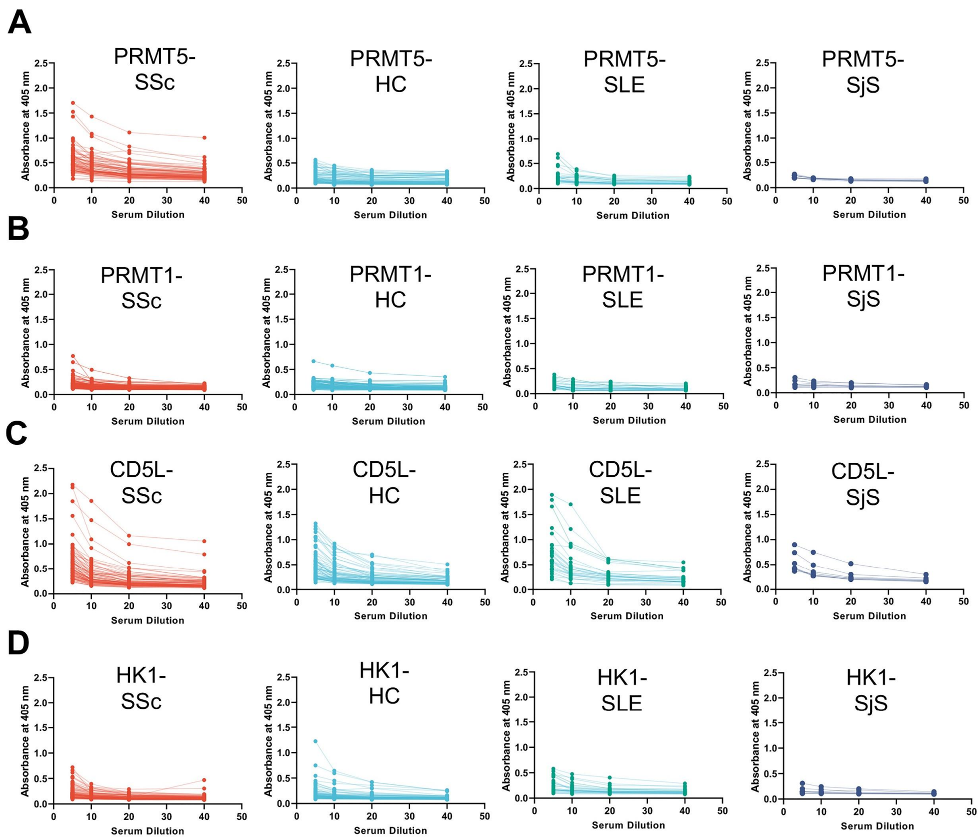

Figure S2. Optimization of dilution for detection of antibodies against PRMT5, PRMT1, CD5L and HK-1.

(**A-D**) Serial dilutions (5×, 10×, 20× and 40×) were performed using serum of the patients with Systemic sclerosis (SSc), Systemic lupus erythematosus (SLE), Sjögren's syndrome (SjS) and healthy controls (HC) by enzyme-linked immunosorbent assay (ELISA) for the assessment of serum levels of antibodies against PRMT5 (A), PRMT1 (B), CD5L (C) and HK-1 (D).

Figure S3

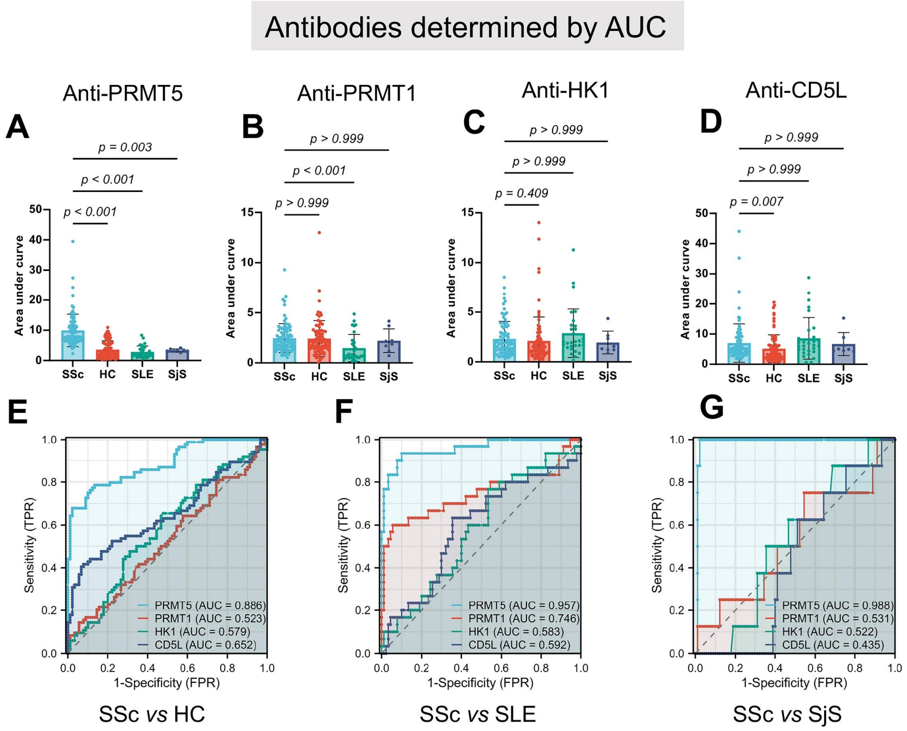

Figure S3. Characterization of serum antibodies against PRMT5, PRMT1, HK-1 and CD5L in the patients of SSc, SLE, SjS and healthy controls.

(**A-D**) Area under curve of antibodies against PRMT5, PRMT1, HK-1 and CD5L in serially diluted serum of SSc, SLE, SjS patients and healthy controls determined by ELISA. (**E-G**) The ROC curves were plotted based on the AUC values, comparing SSc patients with healthy controls (E), SLE patients (F), and SjS patients (G). Comparison data in A-D are shown as bar graphs with individual values, each dot representing one sample, with the median shown as a continuous line and the quartiles as discontinuous lines. *p*-values were determined by Kruskal-Wallis test with Dunn's multiple post hoc test and indicated in the figures.

Liang M, *et al. Ann Rheum Dis* 2024; 83:1144–1155. doi: 10.1136/ard-2024-225596

Figure S4

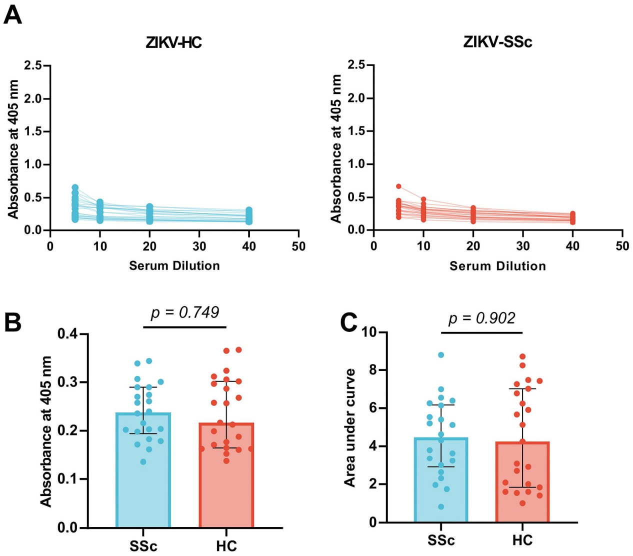

Figure S4. Non-relevant antibodies in SSc determined by ELISA.

(A) Anti-ZIKA virus antibodies, as non-relevant antibodies, were determined in the serum of SSc patients and healthy controls (HC) by ELISA. Serial dilutions (5×, 10×, 20× and 40×) were performed using serum of the 22 patients with SSc and 23 HC. (**B**) Comparison of levels of anti-ZIKA virus antibodies in serum of SSc patients and HC. The signal read at 405 nm with ELISA. (**C**) Comparison of levels of anti-ZIKA virus antibodies in serum of SSc patients and HC. The values calculated by area under curve (AUC) of anti-ZIKA virus antibodies based on serially dilution of serums. Data of B and C are presented as median ± IQR, each dot representing one sample. *p*-values were determined by Mann-Whitney U non-parametric testing for all two-group comparisons. *p*-values are indicated in the figures.

Figure S5

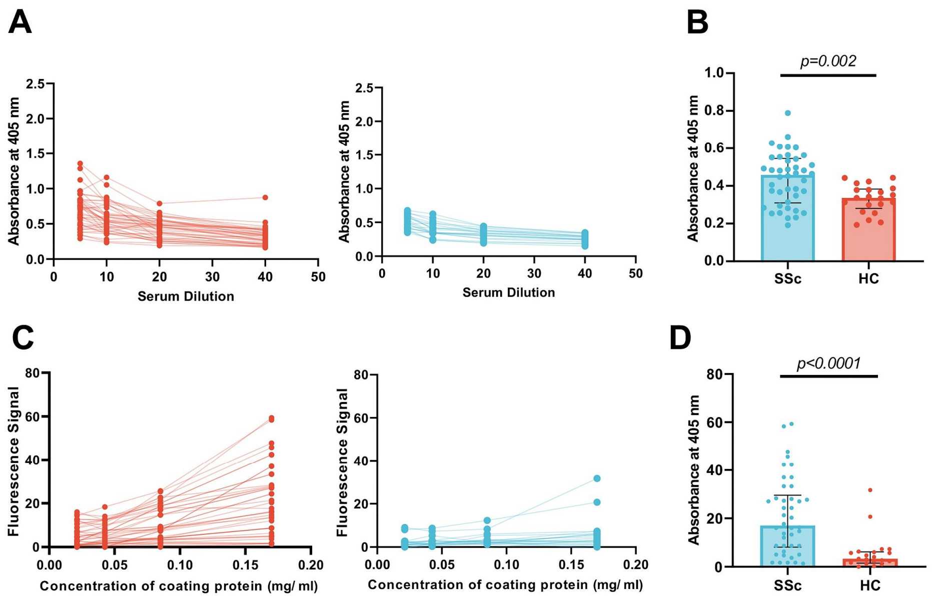

Figure S5. Serum antibodies against PRMT5 in a subsequent validation group of SSc patients.

(A) Serial dilutions (5×, 10×, 20× and 40×) were performed for the assessment of serum levels of antibodies against PRMT5 in the patients with SSc and healthy controls (HC) by enzyme-linked immunosorbent assay (ELISA). (**B**) Comparison of anti-PRMT5 antibodies determined by ELISA, in sera of 42 SSc patients and 21 HC in a 2nd independent validation cohort. (**C**) Antibodies against PRMT5 antibodies determined by microarray, in sera of 42 SSc patients and 21 HC in a 2nd independent validation cohort. The concentrations of recombinant PRMT5 protein printed on microarray slides were 0.17 mg/ml, 0.085 mg/ml, 0.0425 mg/ml, and 0.02125 mg/ml. (**D**) Comparison of anti-PRMT5 antibody levels in sera from SSc patients and HC determined by microarray. Data of B and D are presented as median ± IQR, each dot representing one sample. *p*-values were determined by Mann-Whitney U non-parametric testing for all two-group comparisons. *p*-values are indicated in the figures.

Liang M, *et al. Ann Rheum Dis* 2024; 83:1144–1155. doi: 10.1136/ard-2024-225596

Figure S6

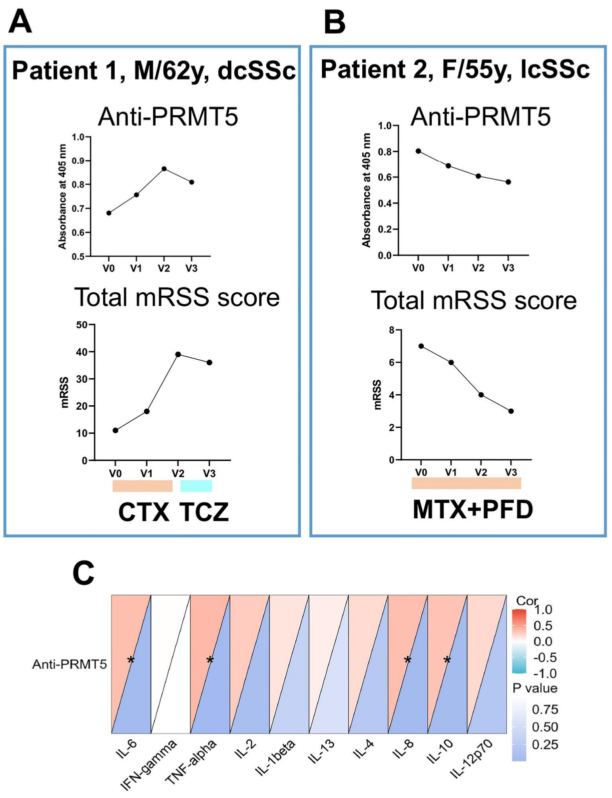

Figure S6. Prospective follow-up of anti-PRMT5 for representative SSc patients after treatment.

Two representative patients underwent a tight follow-up, with assessments of anti-PRMT5 antibody levels and total modified Rodnan Skin Score (mRSS) conducted every 3 months. Baseline measurements were taken, followed by evaluations at the 1st, 2nd, and 3rd visits post-recruitment. (**A**) Patient 1: a 62-year-old male patient, diagnosed with dcSSc, underwent the treatment of Cyclophosphamide (CYC) until the 2nd visit, and transferred to the treatment of Tocilizumab (TCZ) due to less therapeutical response. (**B**) Patient 2: a 55-year-old female patient, diagnosed with lcSSc, underwent the treatment of Methotrexate (MTX) and Pirfenidone (PFD) throughout. (**C**) Heatmap showing correlations between anti-PRMT5 antibody levels and cytokine concentrations (pg/ml) in the sera of SSc patients from the 2nd validation group (n=42). The colors in the upper left square represent Spearman's r value, while the colors in lower right square represent p values (* $p < 0.05$).

Figure S7

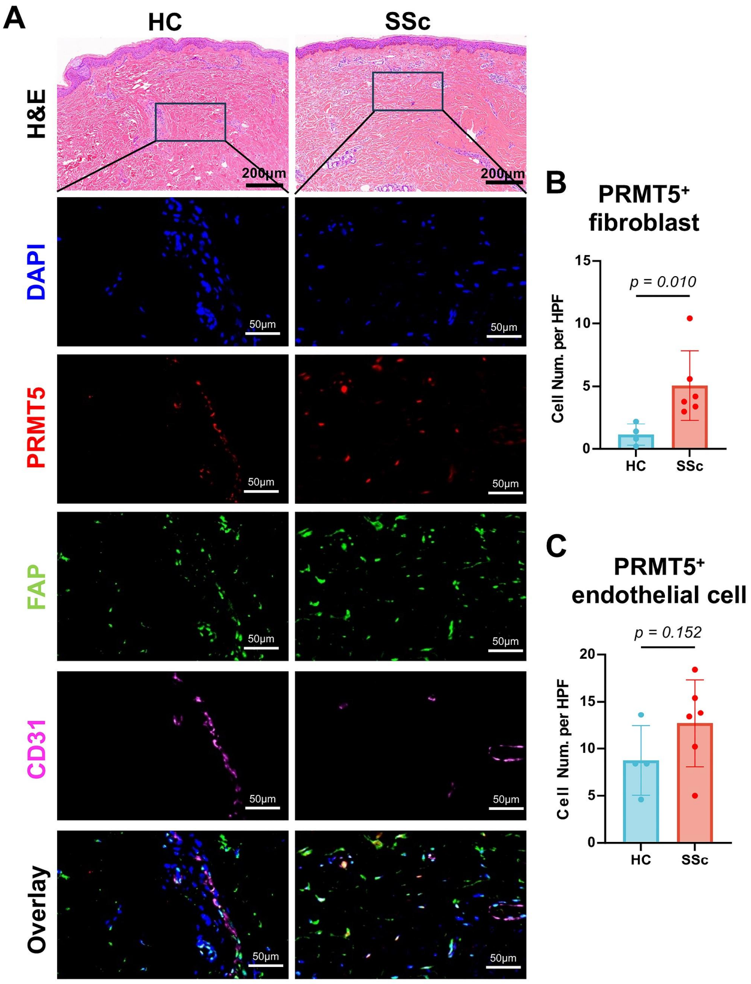

Figure S7. Upregulation of PRMT5 expression in SSc skin fibroblast.

(A) Representative multicolor immunofluorescent stainings for PRMT5 (red), fibroblast marker FAP (green), endothelial cell marker CD31 (pink) and DAPI (blue) in the dermis of healthy donors and SSc patients at 400-fold magnification (scale bars = 50 µm). Hematoxylin and eosin (H&E) stainings (100-fold magnification, scale bars = 200 µm) are included. (**B**) Quantification of numbers of PRMT5-positive fibroblasts per HPF. (**C**) Quantification of numbers of PRMT5-positive endothelial cells per HPF. Data from B and C are presented as median ± IQR, each dot representing one sample. *p*-values were determined by two-sided Mann-Whitney test, and the *p*-values are indicated in the figures (n = 6 SSc patients and n = 4 healthy individuals).

Liang M, *et al. Ann Rheum Dis* 2024; 83:1144–1155. doi: 10.1136/ard-2024-225596

Figure S8

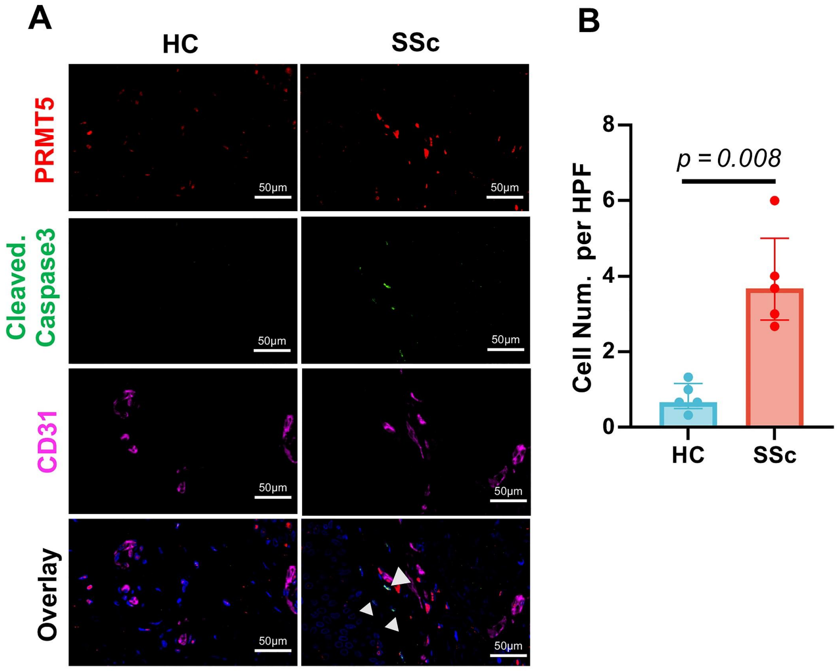

Figure S8. PRMT5 expression in apoptotic endothelial cells in SSc skin. Representative multicolor immunofluorescent stainings for PRMT5 (red), endothelial cleaved caspase3 (green), cell marker CD31 (pink), and DAPI (blue) in the dermis of healthy donors and SSc patients at 400-fold magnification (scale bars = 50 μm). The presence of Cleaved Caspase3$^+$PRMT5$^+$ endothelial cells is highlighted by arrows. Quantification of numbers of Cleaved Caspase3$^+$PRMT5$^+$CD31$^+$ endothelial cells per high-power field (HPF). Data from B are presented as median ± IQR, each dot representing one sample. *p*-values were determined by two-sided Mann-Whitney test, and the *p*-values are indicated in the figures (n = 5 SSc patients and n = 5 healthy individuals).

Liang M, *et al. Ann Rheum Dis* 2024; 83:1144–1155. doi: 10.1136/ard-2024-225596

Figure S9

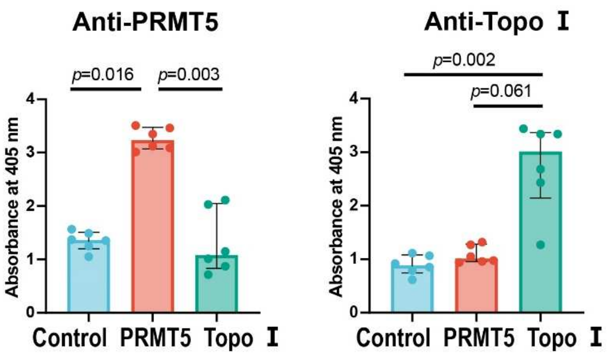

Figure S9. Serum antibody response to PRMT5 in mice.

Detection of antibody response to PRMT5 or DNA topoisomerase I (Topo I) in sera of immunized mice by ELISA. C57BL/6 mice were immunized with recombinant PRMT5 or Topo I protein, each in conjunction with complete Freund's adjuvant (CFA), administered at 2-week intervals for a total of 4 immunizations. The sera were collected and subjected to ELISA to assess the antibody response to PRMT5 or Topo I with a serum dilution of 1:10. Data are presented as median ± IQR, each dot representing one sample. *p*-values were determined by Kruskal-Wallis test with Dunn's multiple post hoc tests. *p*-values are indicated in the figures. n = 6 independent biological samples per group.

Liang M, *et al. Ann Rheum Dis* 2024; 83:1144–1155. doi: 10.1136/ard-2024-225596

Figure S10

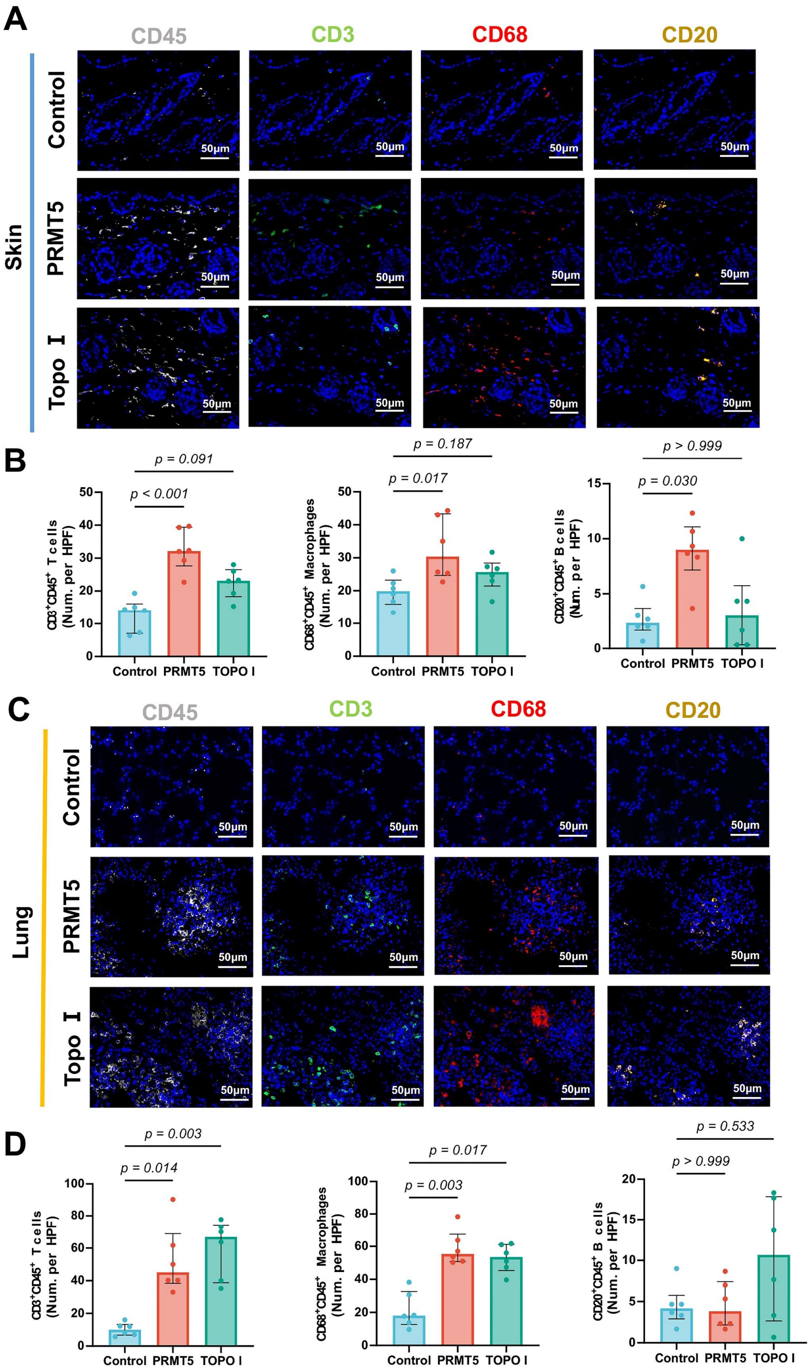

Figure S10. Induction of immune infiltration in mice immunized by recombinant protein PRMT5.

(A) Immunofluorescent staining for CD45 (grey), CD3 (green), CD68 (red), CD20 (gold) and DAPI (blue) in the skin of mice immunized with recombinant PRMT5, or

DNA topoisomerase I (Topo I), along with complete Freund's adjuvant (CFA), at 400-fold magnification. (**B**) Quantification of numbers of $CD3^+$ T cells, $CD68^+$ macrophages and $CD20^+$ B cells from all $CD45^+$ immune cells in the skin (n = 6 independent biological samples per group). Scale bars = 50 μm. (**C**) Immunofluorescent stainings for CD45 (grey), CD3 (green), CD68 (red), CD20 (gold) and DAPI (blue) in the lungs of mice immunized with recombinant PRMT5, or Topo I, along with CFA, at 400-fold magnification. (**D**) Quantification of numbers of $CD3^+$ T cells, $CD68^+$ macrophages and $CD20^+$ B cells from all $CD45^+$ immune cells in the lungs (n = 6 independent biological samples per group). Scale bars = 50 μm. All data are presented as median ± interquartile range (IQR), each dot representing one sample. *p*-values were determined by Kruskal-Wallis test with Dunn's multiple post hoc test (B, D). *p*-values are indicated in the figures.

Liang M, *et al. Ann Rheum Dis* 2024; 83:1144–1155. doi: 10.1136/ard-2024-225596

Figure S11

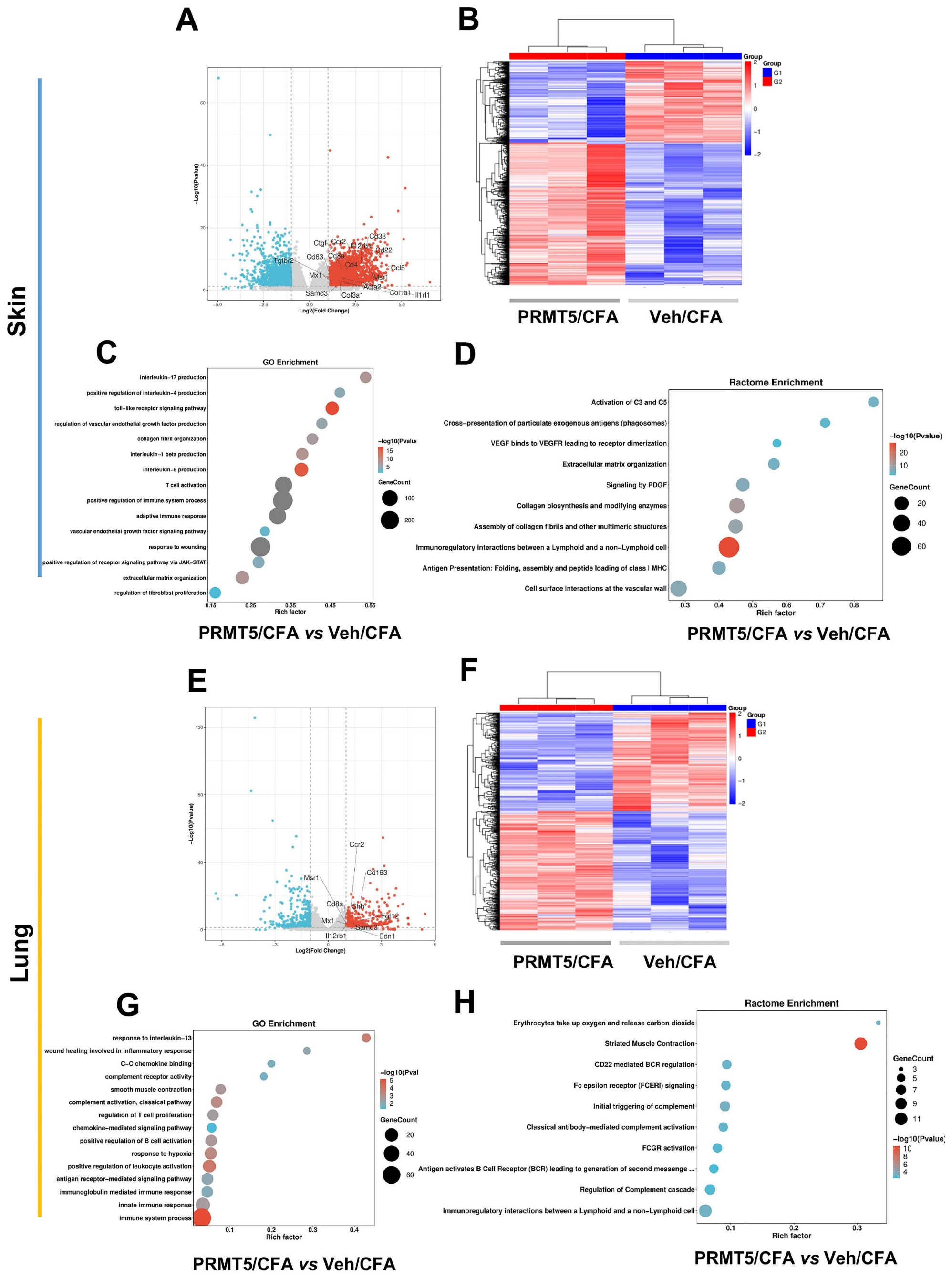

Figure S11: Immunization with recombinant protein PRMT5 results in multifaceted proinflammatory and profibrotic response in the skin and lungs of mice.

RNA sequencing (RNA-seq) was conducted on the skin and lung tissues from mice

Liang M, *et al. Ann Rheum Dis* 2024; 83:1144–1155. doi: 10.1136/ard-2024-225596

immunized with PRMT5 alongside adjuvant complete Freund's adjuvant (CFA) (PRMT5 / CFA group), in comparison to the mice injected with vehicle and CFA (vehicle / CFA group) as control (n = 3 independent biological samples per group). (**A**, **E**) The volcano plots showing DEGs in the skin (A) and lungs (E) of mice comparing between PRMT5 / CFA group and vehicle / CFA group. The volcano plot in A showing a total of 4205 DEGs in the skin of PRMT5 / CFA-treated mice compared to vehicle / CFA-treated control mice (red dots: 2681 upregulated DEGs, blue dots: 1524 downregulated DEGs), with the thresholds of p-value < 0.05 and $|\log2FC| \geq 1$. The volcano plot in E showing a total of 1169 DEGs in the PRMT5 / CFA-treated mice compared to vehicle / CFA-treated control mice (red dots: 640 upregulated DEGs, blue dots: 529 downregulated DEGs), with the thresholds of p-value < 0.05 and $|\log2FC| \geq 1$. (**B**, **F**) Hierarchical cluster and gene heatmap illustrating the gene expression profiles of the DEGs in skin (B) and lungs (F), comparing between PRMT5 / CFA-treated mice with the vehicle / CFA-treated control mice. (**C**, **G**) Dot plots displaying enrichment of the significant Gene Ontology (GO) biological processes relevant to fibrosis and inflammation among top terms in the skin (C) and lungs (G) from PRMT5 / CFA-treated mice, compared to vehicle / CFA-treated control mice. The colors of the dots represent -log10 (p values), and the size of the dots represents the number of genes in the DEGs in the datasets associated with the GO term. (**D**, **H**) Enriched biological pathways analyses by Reactome in skin (D) and lungs (H), comparing between PRMT5 / CFA-treated mice with vehicle / CFA-treated control mice. In C, D, G and H, the color indicates the -log10 (p values), and the bubble size indicates the number of genes annotated to the pathway in the reference database. The rich factor represents the ratio of the total number of genes belonging to the pathway to the total number of genes.

Figure S12

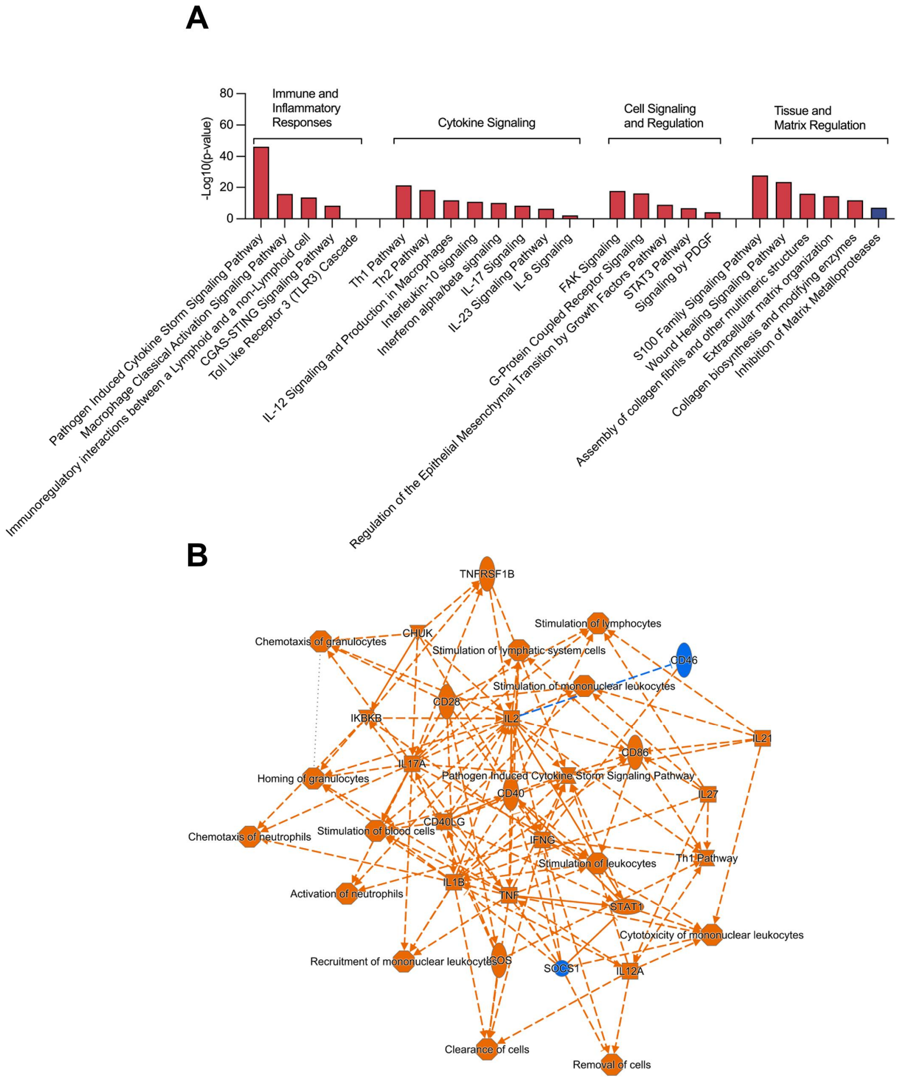

Figure S12. Ingenuity Pathway Analysis (IPA) for the signaling pathways influenced by differentially expressed genes (DEGs) between the mice immunized with PRMT5 / CFA and vehicle / CFA.

(**A**) Corresponding canonical signaling pathways regulated by the DEGs in skin tissue. z score is computed and used to reflect the predicted activation level (z > 0, activated; z < 0, inhibited; ≥ 2 or ≤ −2 can be considered significant). (**B**) Graphical network of canonical pathways, upstream regulators, and biological functions regulated by DEGs identified in skin tissue.

Table S1. Clinical features of SSc patients who underwent antibody exploration

Characteristics	Patient 1	Patient 2	Patient 3
Sex (female/male)	m	m	f
Age at recruitment, years	62	67	29
Subsets (dcSSc/lcSSc)	dcSSc	dcSSc	dcSSc
Disease duration from first non-RP, months	8	10	18
Disease duration (early/late) *	early	early	early
Total mRSS †	31	18	8
Digital ulcer (s)	no	no	no
Digital Pitting Scar (s)	yes	no	no
Arthralgia	yes	yes	no
Myalgia	yes	no	no
Telangiectasia	yes	no	no
Interstitial lung disease (ILD) §	yes	yes	no
Pulmonary arterial hypertension (PAH)	no	no	no
CRP (mg/L)	6.45	3.7	1.6
ESR (mm/h)	27	8	24
IgG (g/L)	19.9	12.7	14.3
C3 (g/L)	0.87	0.71	0.81
C4 (g/L)	0.30	0.18	0.20
**Disease specific autoantibodies**			
ATA positive	+		+
ACA positive			
ARA positive		+	

Liang M, *et al. Ann Rheum Dis* 2024; 83:1144–1155. doi: 10.1136/ard-2024-225596

BMJ Publishing Group Limited (BMJ) disclaims all liability and responsibility arising from any reliance placed on this supplemental material which has been supplied by the author(s)

Table S2. Clinical features of SSc patients who initially underwent antibody validation

Characteristics	n=90
Recruiting period	Oct 01, 2018~Oct 01, 2020
Sex (female/male)	72/18 (80% / 20%)
Age at recruitment, years	50.08 ± 13.23
Subsets (dcSSc/lcSSc)	48/42 (53.33% / 46.67%)
Disease duration from first non-RP, years	4.116 ± 4.862
Disease duration (early/late) *	56/34 (62.22% / 37.78%)
Total mRSS †	11.50 ± 10.35
Digital ulcer (s)	27 (30.00%)
Digital Pitting Scar (s)	32 (35.56%)
Arthralgia	32 (35.56%)
Myalgia	23 (25.56%)
Telangiectasia	46 (51.11%)
Interstitial lung disease (ILD) §	54 (60.00%)
Pulmonary arterial hypertension (PAH)	4 (4.44%)
CRP (mg/L)	5.89 ± 9.763
ESR (mm/h)	27.24 ± 18
IgG (g/L)	14.28 ± 7.055
C3 (g/L)	0.8455 ± 0.1490
C4 (g/L)	0.1919 ± 0.05892
Disease specific autoantibodies	
ATA positive	45 (50%)
ACA positive	21 (23.33%)
ARA positive	6 (6.67%)

Table S3. Clinical features of SSc patients who subsequently underwent antibody validation

Characteristics	n=42
Recruiting period	Oct 01, 2022~Feb 01, 2024
Sex (female/male)	30/12 (71% / 29%)
Age at recruitment, years	56.43 ± 13.19
Subsets (dcSSc/lcSSc)	19/22 (46.34% / 53.66%)
Disease duration from first non-RP, years	3.543 ± 3.880
Disease duration (early/late) *	26/16 (61.90% / 39.02%)
Total mRSS †	13.31 ± 10.18
Digital ulcer (s)	13 (31.71%)
Digital Pitting Scar (s)	11 (26.19%)
Arthralgia	14 (33.33%)
Myalgia	23 (25.56%)
Telangiectasia	19 (45.24%)
Interstitial lung disease (ILD) §	22 (52.38%)
Pulmonary arterial hypertension (PAH)	2 (4.88%)
CRP (mg/L)	6.26 ± 7.50
ESR (mm/h)	30.07 ± 31.45
IgG (g/L)	13.77 ± 6.45
C3 (g/L)	0.9218 ± 0.1639
C4 (g/L)	0.2125 ± 0.07276
Disease specific autoantibodies	
ATA positive	16 (38.10%)
ACA positive	12 (28.57 %)
ARA positive	10 (23.81%)

* as categorized by the duration from first non-RP symptoms (early ≤ 3 years; late > 3 years); † as referred to the total mRSS assessed at 17 anatomic sites; § as detected by lung high-resolution CT;

Liang M, *et al. Ann Rheum Dis* 2024; 83:1144–1155. doi: 10.1136/ard-2024-225596

Abbreviations: SSc, systemic sclerosis; dcSSc, diffuse cutaneous systemic sclerosis; lcSSc, limited cutaneous systemic sclerosis; RP, Raynaud's phenomenon; mRSS, modified Rodnan skin score; ILD, interstitial lung disease; PAH, pulmonary arterial hypertension; CRP, C-reactive protein; ESR, Erythrocyte Sedimentation Rate; IgG, Immunoglobulin G (IgG); C3, complement 3; C4, complement 4; ATA, anti-topoisomerase I antibody; ACA, anti-centromere antibody; ARA, anti-RNA polymerase III antibody

Table S4. Information of recombinant proteins applied in Enzyme-Linked Immunosorbent Assay (ELISA) and microarray

Protein	Abbreviation	Source	Identifier	Species	Sequence
Protein Arginine Methyltransferase 5	PRMT5	Sino Biological	Cat# 11074-H32B	Human	Ala2-Leu637
Protein Arginine Methyltransferase 1	PRMT1	Abcam	Cat# Ab89007	Human	Full length
Hexokinase 1	HK-1	Abcam	Cat# Ab85918	Human	Full length
CD5L	CD5L	Novoprotein	Cat# C580	Human	Ser20-Gly347
ZIKA virus domain III	/	Self-produced	/	ZIKV strain MR766	303-404

Reference

1. Zhou F, Lu Y, Ficarro SB, et al. Genome-scale proteome quantification by DEEP SEQ mass spectrometry. *Nat Commun* 2013;4:2171. doi: 10.1038/ncomms3171
2. van den Hoogen F, Khanna D, Fransen J, et al. 2013 classification criteria for systemic sclerosis: an American college of rheumatology/European league against rheumatism collaborative initiative. *Ann Rheum Dis* 2013;72(11):1747-55. doi: 10.1136/annrheumdis-2013-204424 [published Online First: 2013/10/05]
3. LeRoy EC, Black C, Fleischmajer R, et al. Scleroderma (systemic sclerosis): classification, subsets and pathogenesis. *The Journal of rheumatology* 1988;15(2):202-5.
4. Ichikado K, Suga M, Muranaka H, et al. Prediction of prognosis for acute respiratory distress syndrome with thin-section CT: validation in 44 cases. *Radiology*

2006;238(1):321-9. doi: 10.1148/radiol.2373041515

5. Cottin V, Hirani NA, Hotchkin DL, et al. Presentation, diagnosis and clinical course of the spectrum of progressive-fibrosing interstitial lung diseases. *European respiratory review : an official journal of the European Respiratory Society* 2018;27(150) doi: 10.1183/16000617.0076-2018
6. Khanna D, Lin CJF, Furst DE, et al. Long-Term Safety and Efficacy of Tocilizumab in Early Systemic Sclerosis-Interstitial Lung Disease: Open-Label Extension of a Phase 3 Randomized Controlled Trial. *Am J Respir Crit Care Med* 2022;205(6):674-84. doi: 10.1164/rccm.202103-0714OC
7. Liu X, Zhang Y, Chen Y, et al. In Situ Capture of Chromatin Interactions by Biotinylated dCas9. *Cell* 2017;170(5):1028-43 e19. doi: 10.1016/j.cell.2017.08.003
8. Li M, Tu S, Li Z, et al. MAP: model-based analysis of proteomic data to detect proteins with significant abundance changes. *Cell Discov* 2019;5:40. doi: 10.1038/s41421-019-0107-9 [published Online First: 20190813]
9. Wu Y, Li S, Du L, et al. Neutralization of Zika virus by germline-like human monoclonal antibodies targeting cryptic epitopes on envelope domain III. *Emerg Microbes Infect* 2017;6(10):e89. doi: 10.1038/emi.2017.79 [published Online First: 20171011]
10. Jiang HW, Li Y, Zhang HN, et al. SARS-CoV-2 proteome microarray for global profiling of COVID-19 specific IgG and IgM responses. *Nat Commun* 2020;11(1):3581. doi: 10.1038/s41467-020-17488-8 [published Online First: 20200714]
11. Yoshizaki A, Yanaba K, Ogawa A, et al. Immunization with DNA topoisomerase I and Freund's complete adjuvant induces skin and lung fibrosis and autoimmunity via interleukin-6 signaling. *Arthritis Rheum* 2011;63(11):3575-85. doi: 10.1002/art.30539
12. Zehender A, Li YN, Lin NY, et al. TGFbeta promotes fibrosis by MYST1-dependent epigenetic regulation of autophagy. *Nature communications* 2021;12(1):4404. doi: 10.1038/s41467-021-24601-y
13. Liang M, Lv J, Zou L, et al. A modified murine model of systemic sclerosis: bleomycin given by pump infusion induced skin and pulmonary inflammation and fibrosis. *Lab Invest* 2015;95(3):342-50. doi: 10.1038/labinvest.2014.145 [published Online First: 2014/12/17]
14. Liang M, Lv J, Jiang Z, et al. Promotion of Myofibroblast Differentiation and Tissue Fibrosis by the Leukotriene B(4) -Leukotriene B(4) Receptor Axis in Systemic Sclerosis. *Arthritis Rheumatol* 2020;72(6):1013-25. doi: 10.1002/art.41192 [published Online First: 20200430]
15. Huang J, Beyer C, Palumbo-Zerr K, et al. Nintedanib inhibits fibroblast activation and ameliorates fibrosis in preclinical models of systemic sclerosis. *Annals of the rheumatic diseases* 2016;75(5):883-90. doi: 10.1136/annrheumdis-2014-207109
16. Hubner RH, Gitter W, El Mokhtari NE, et al. Standardized quantification of pulmonary fibrosis in histological samples. *BioTechniques* 2008;44(4):507-11,

14-7. doi: 10.2144/000112729

17. Stack EC, Wang C, Roman KA, Hoyt CC. Multiplexed immunohistochemistry, imaging, and quantitation: a review, with an assessment of Tyramide signal amplification, multispectral imaging and multiplex analysis. *Methods* 2014;70(1):46-58. doi: 10.1016/j.ymeth.2014.08.016 [published Online First: 20140919]
