# Supplementary figures and images for: Identification and validation of anti-protein arginine methyltransferase 5 (PRMT5) antibody as a novel biomarker for systemic sclerosis (SSc)
Source: Ann Rheum Dis. 2024 Apr 29;83(9):1144–55. doi: 10.1136/ard-2024-225596 (PMC11420721; doi:10.1136/ard-2024-225596)

Figure S1

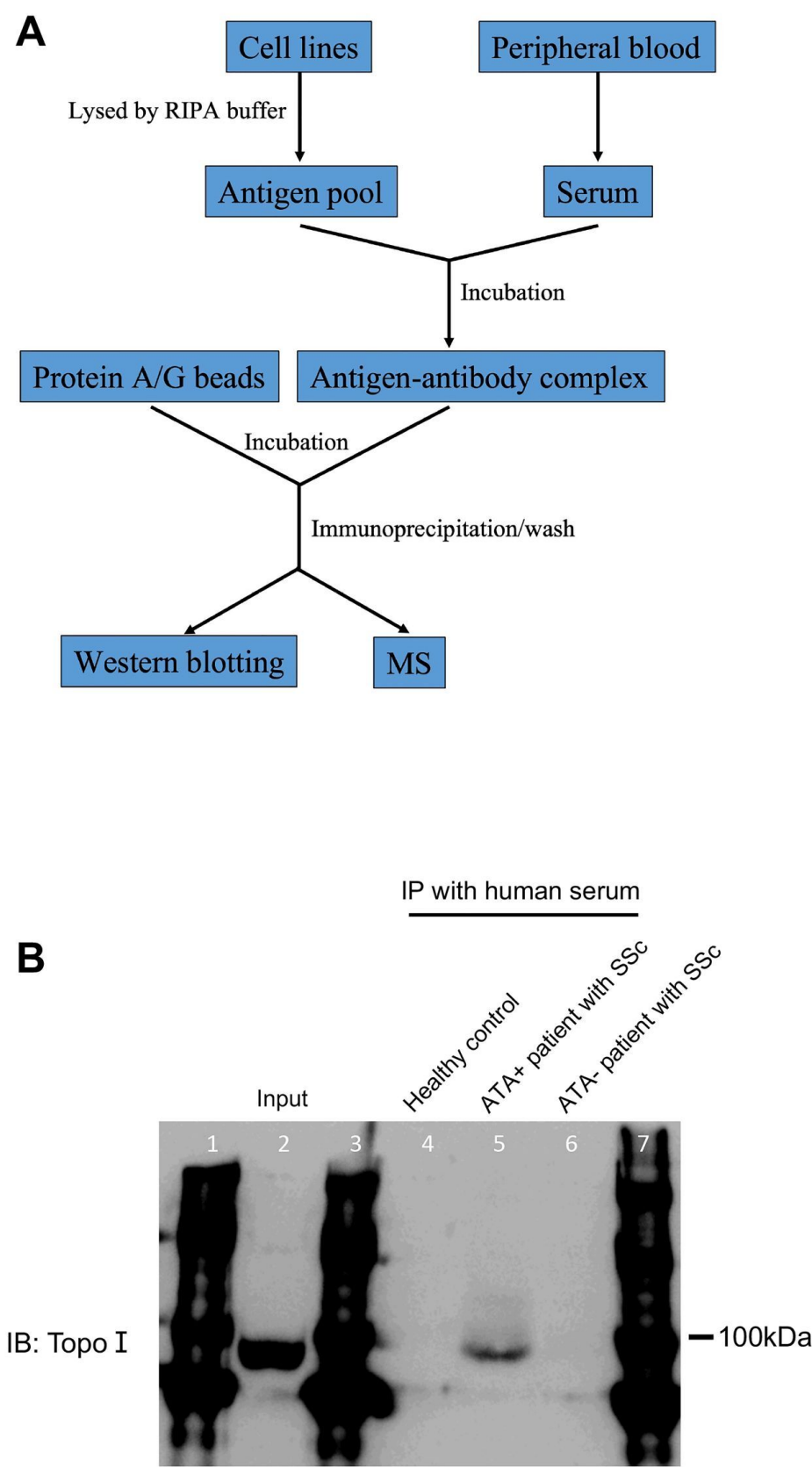

Supplement: Supplementary data [file ard-2024-225596supp001.pdf]

Figure S2

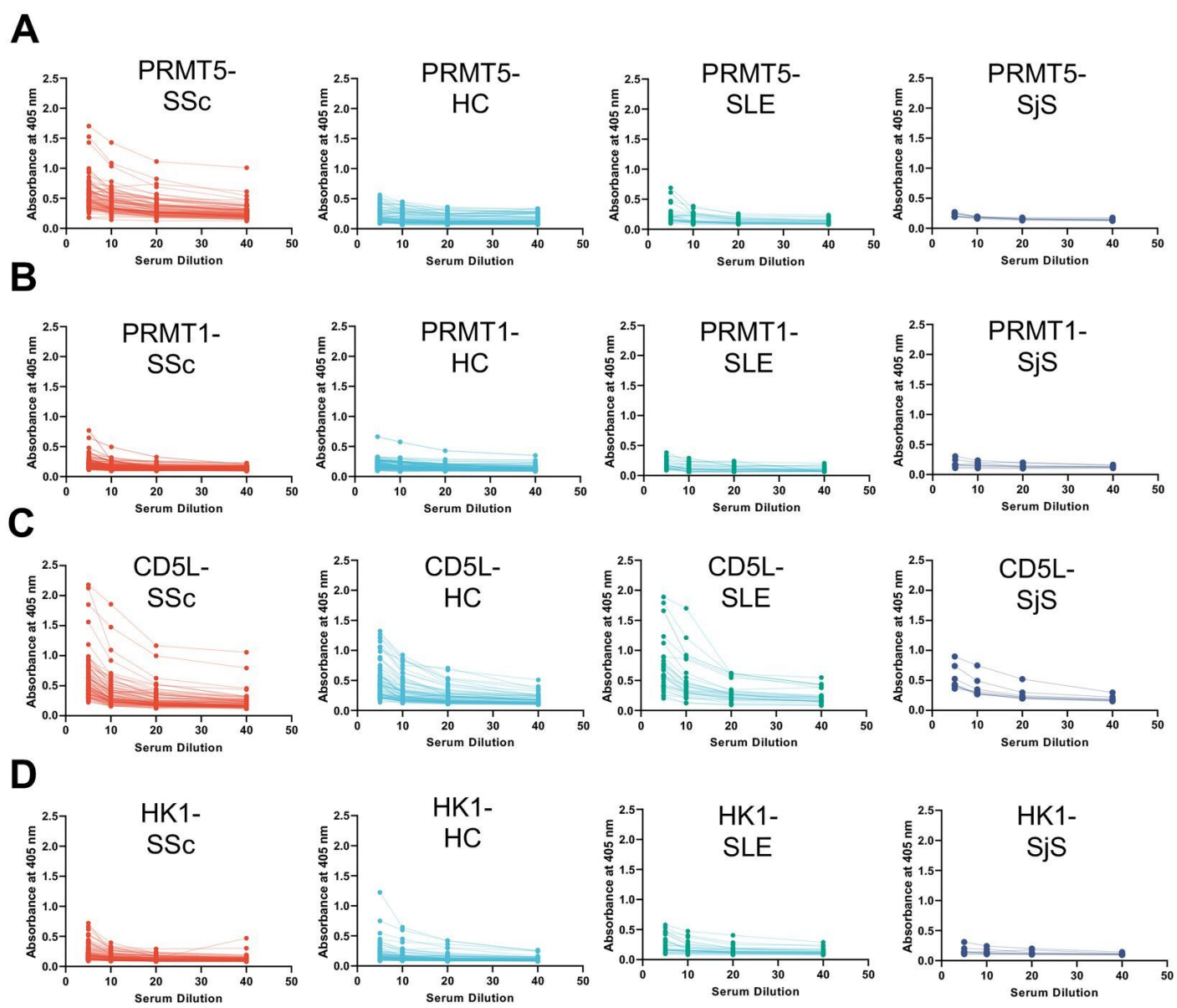

Supplement: Supplementary data [file ard-2024-225596supp002.pdf]

Figure S3

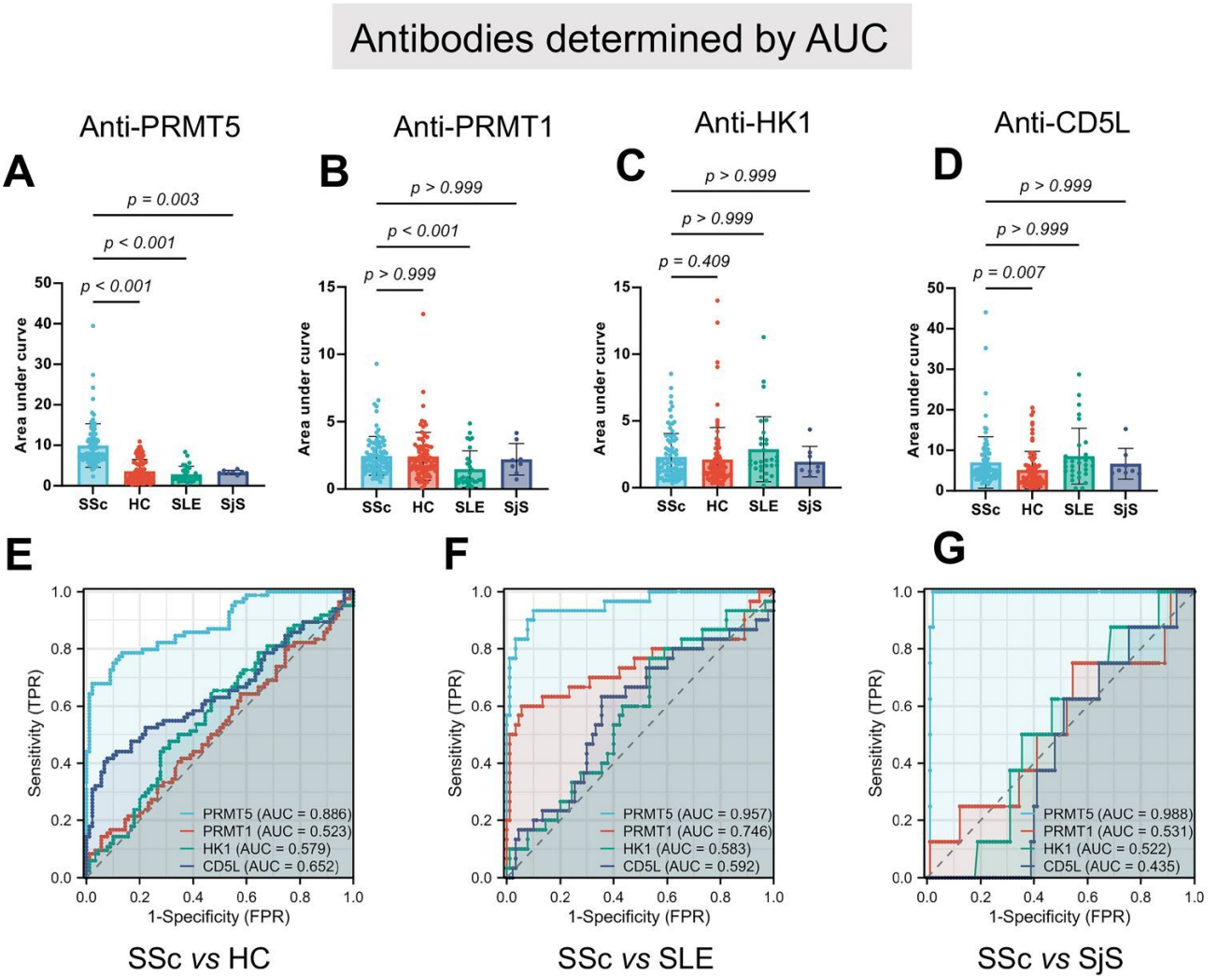

Supplement: Supplementary data [file ard-2024-225596supp003.pdf]

Figure S4

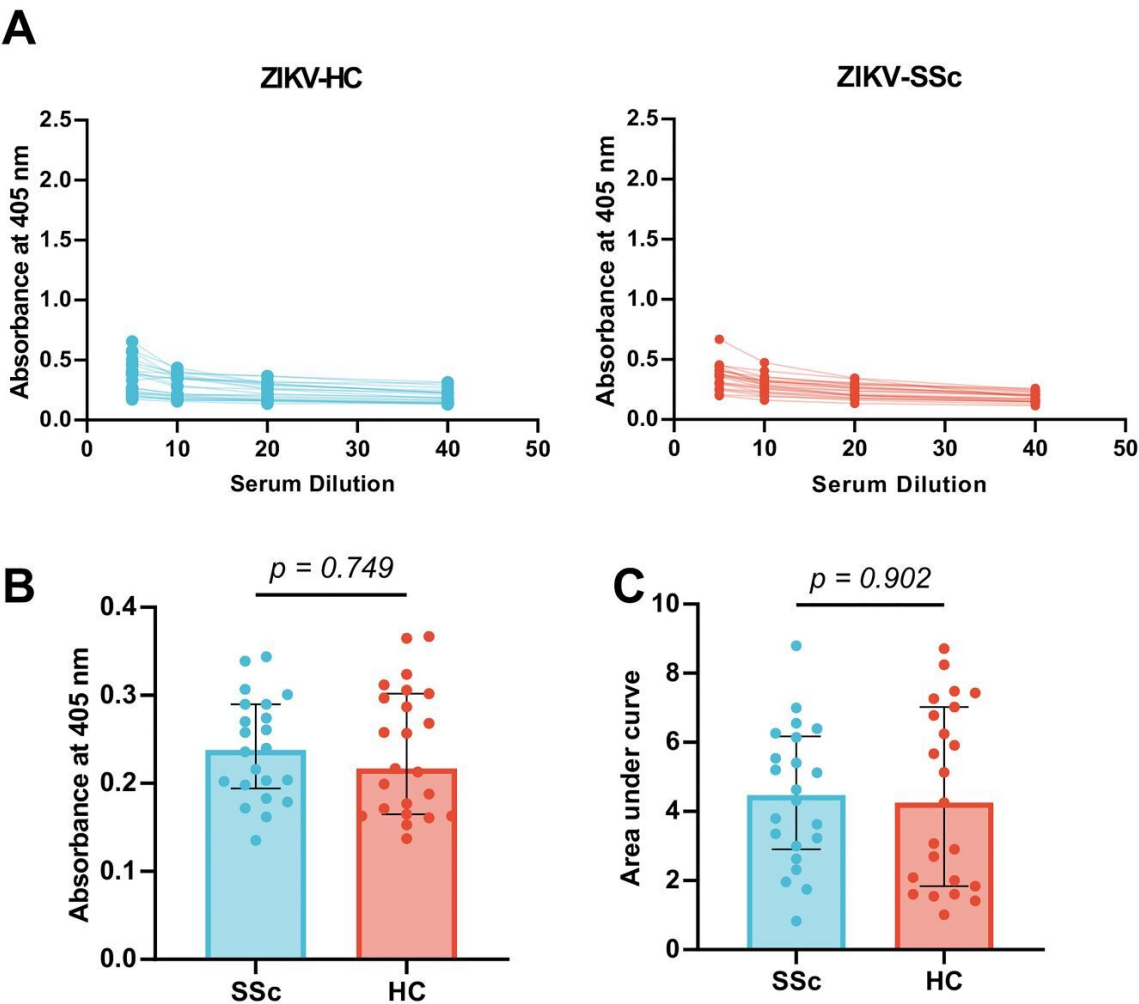

Supplement: Supplementary data [file ard-2024-225596supp004.pdf]

Figure S5

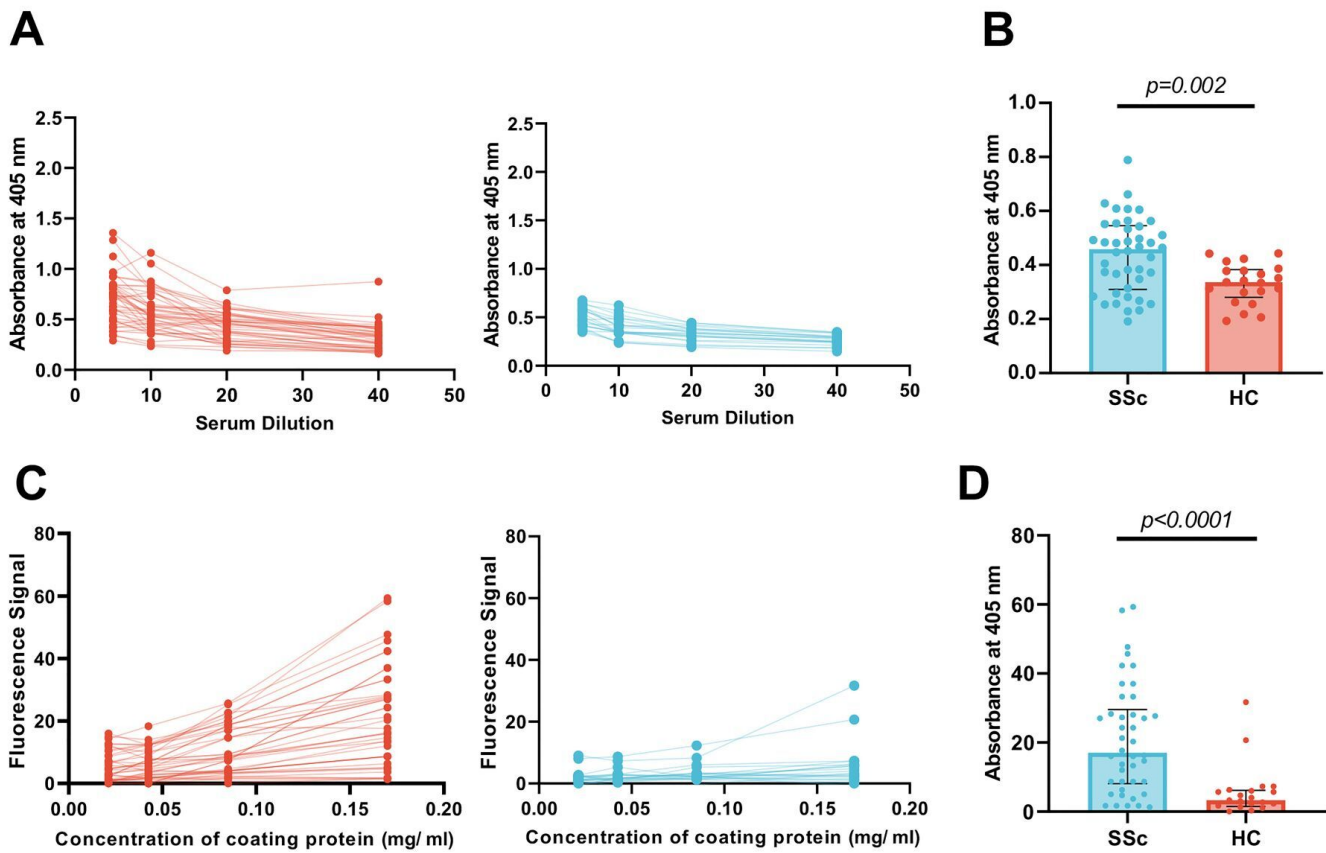

Supplement: Supplementary data [file ard-2024-225596supp005.pdf]

Figure S6

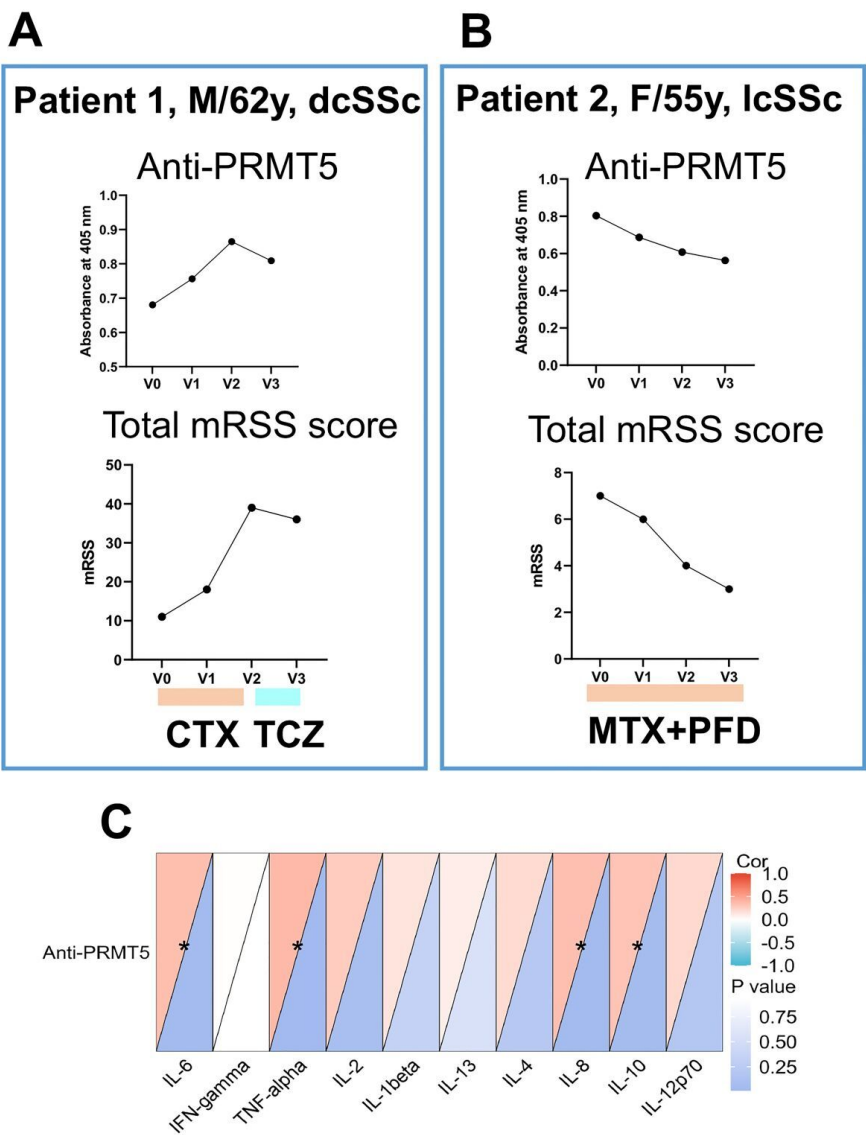

Supplement: Supplementary data [file ard-2024-225596supp006.pdf]

Figure S7

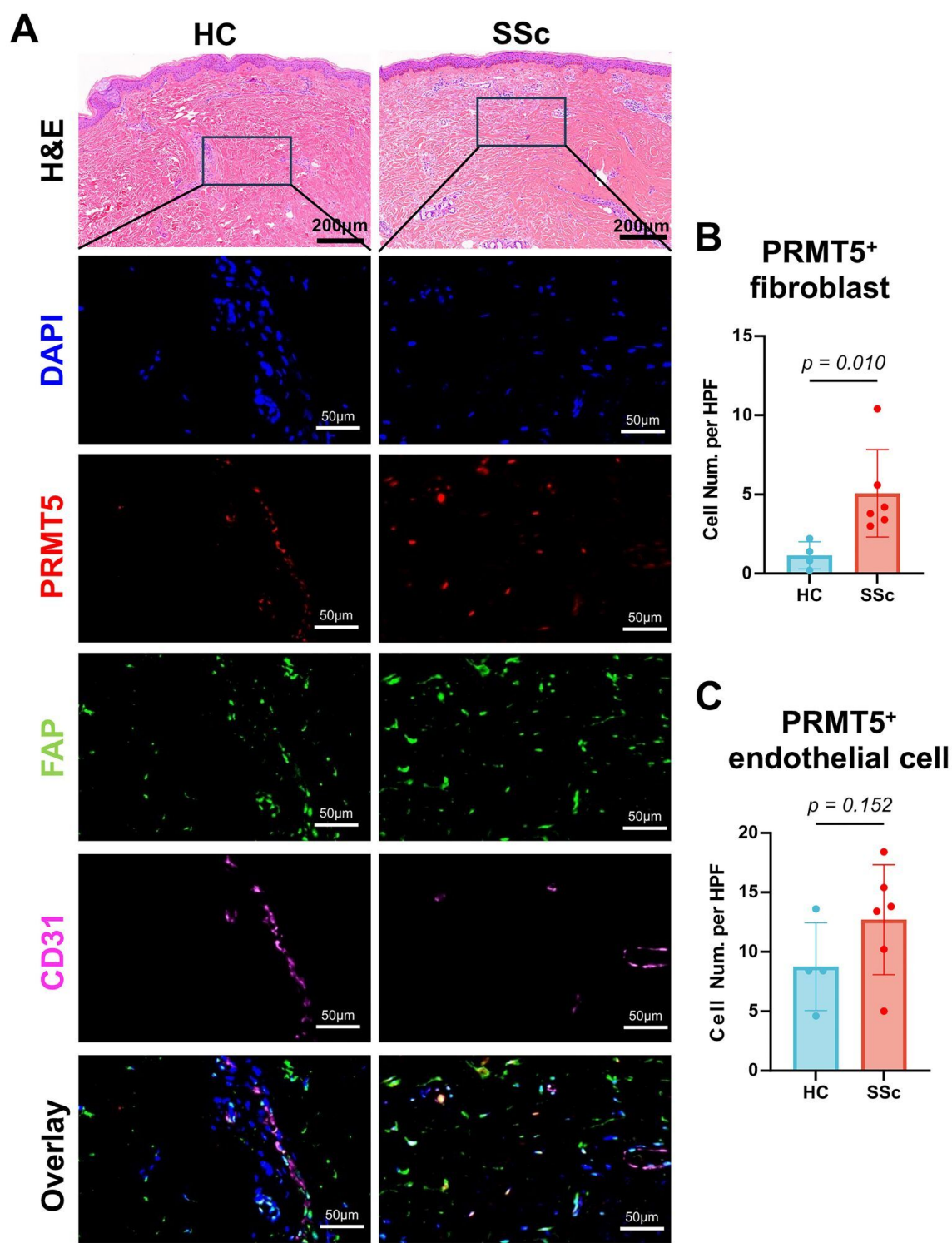

Supplement: Supplementary data [file ard-2024-225596supp007.pdf]

Figure S8

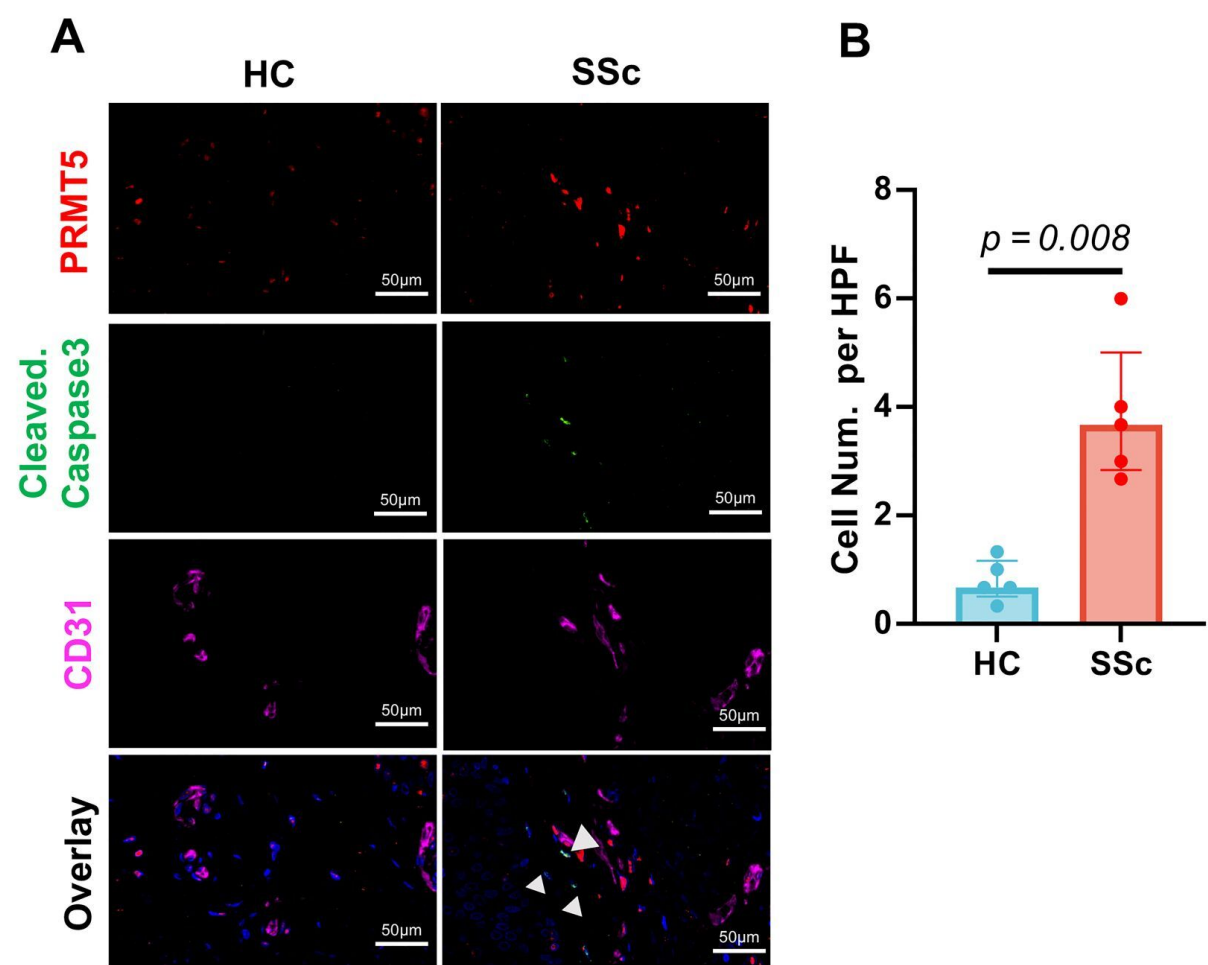

Supplement: Supplementary data [file ard-2024-225596supp008.pdf]

Figure S9

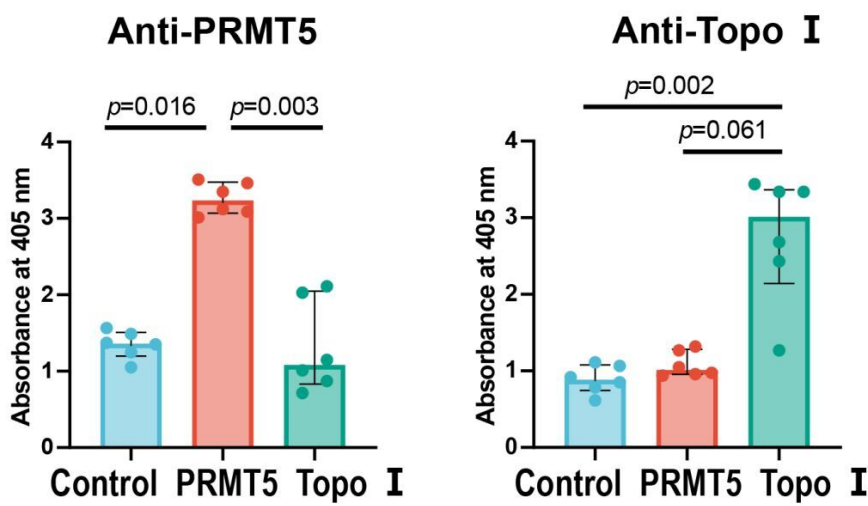

Supplement: Supplementary data [file ard-2024-225596supp009.pdf]

Figure S10

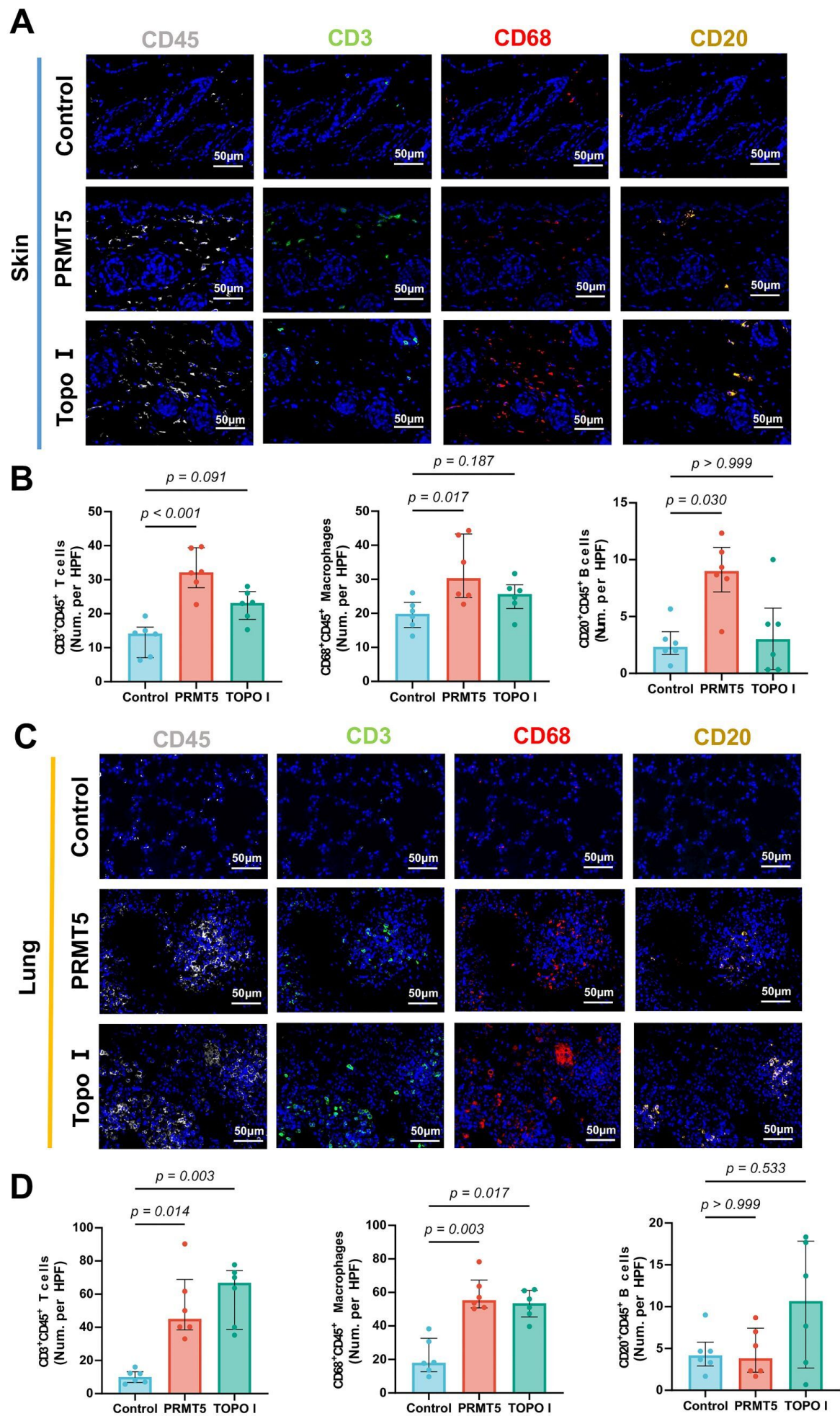

Supplement: Supplementary data [file ard-2024-225596supp010.pdf]

**Figure S11**

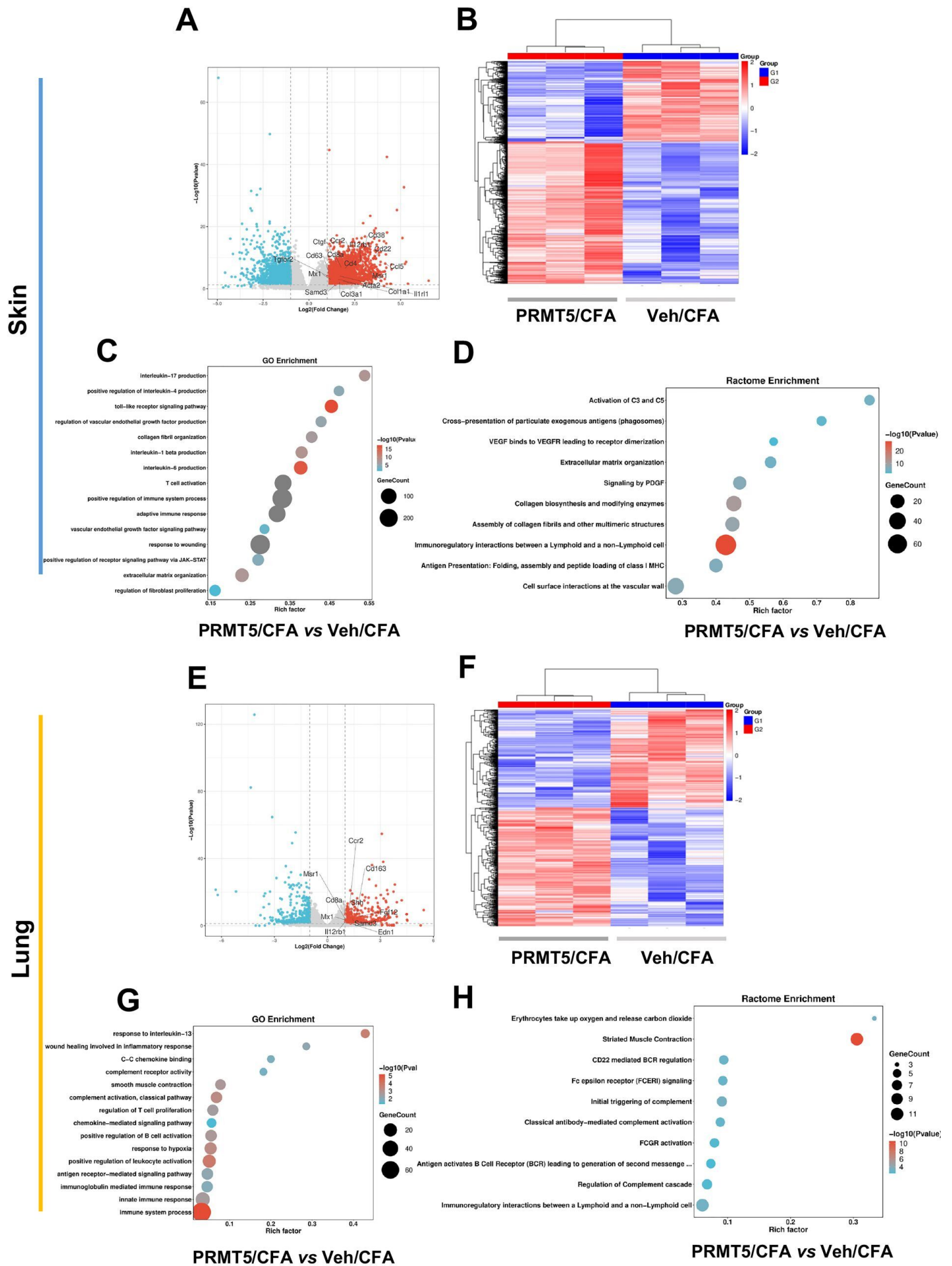

Supplement: Supplementary data [file ard-2024-225596supp011.pdf]

**A**

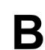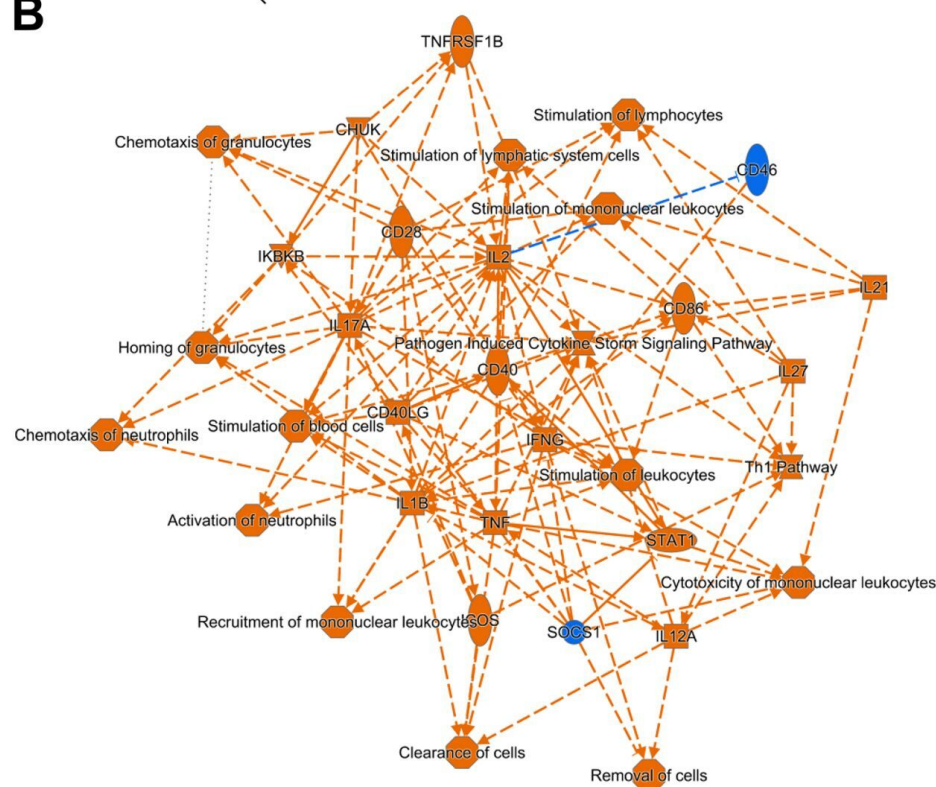

Supplement: Supplementary data [file ard-2024-225596supp012.pdf]
